# Implementation and Impacts of Surface and Blowing Snow Sources of Arctic Bromine Activation Within WRF‐Chem 4.1.1

**DOI:** 10.1029/2020MS002391

**Published:** 2021-07-30

**Authors:** Louis Marelle, Jennie L. Thomas, Shaddy Ahmed, Katie Tuite, Jochen Stutz, Aurélien Dommergue, William R. Simpson, Markus M. Frey, Foteini Baladima

**Affiliations:** ^1^ Institut des Géosciences de l'Environnement, de l'Université Grenoble Alpes, CNRS, IRD, Grenoble INP Grenoble France; ^2^ LATMOS/IPSL Sorbonne Université UVSQ CNRS Paris France; ^3^ Department of Atmospheric and Oceanic Sciences University of California Los Angeles CA USA; ^4^ Geophysical Institute and Department of Chemistry and Biochemistry University of Alaska Fairbanks Fairbanks AK USA; ^5^ British Antarctic Survey Natural Environment Research Council Cambridge UK

**Keywords:** aerosol chemistry, arctic ozone, atmospheric chemistry, halogen chemistry, snow emissions

## Abstract

Elevated concentrations of atmospheric bromine are known to cause ozone depletion in the Arctic, which is most frequently observed during springtime. We implement a detailed description of bromine and chlorine chemistry within the WRF‐Chem 4.1.1 model, and two different descriptions of Arctic bromine activation: (1) heterogeneous chemistry on surface snow on sea ice, triggered by ozone deposition to snow (Toyota et al., 2011 https://doi.org/10.5194/acp-11-3949-2011), and (2) heterogeneous reactions on sea salt aerosols emitted through the sublimation of lofted blowing snow (Yang et al., 2008, https://doi.org/10.1029/2008gl034536). In both mechanisms, bromine activation is sustained by heterogeneous reactions on aerosols and surface snow. Simulations for spring 2012 covering the entire Arctic reproduce frequent and widespread ozone depletion events, and comparisons with observations of ozone show that these developments significantly improve model predictions during the Arctic spring. Simulations show that ozone depletion events can be initiated by both surface snow on sea ice, or by aerosols that originate from blowing snow. On a regional scale, in spring 2012, snow on sea ice dominates halogen activation and ozone depletion at the surface. During this period, blowing snow is a major source of Arctic sea salt aerosols but only triggers a few depletion events.

## Key Points

- Halogen activation and its role in Arctic surface ozone depletion events (ODEs) is modeled using WRF‐Chem
- Two halogen activation mechanisms are implemented (1) surface snow and (2) blowing snow
- A spring 2012 case study indicates that both mechanisms can trigger near‐surface ODEs, but that surface snow dominates

## 1 Introduction

During Arctic spring, the Atmospheric Boundary Layer (ABL), experiences episodic depletion of ozone to values less than 10 parts per billion by volume (ppbv), far below background levels of ∼40 ppbv (Barrie et al., 1988; Oltmans, 1981). These well‐known ozone depletion events (ODEs) are tied to the presence of enhanced concentrations of reactive bromine in the atmosphere (Barrie et al., 1988), including species such as Br_2_, BrO, Br, HOBr, and BrNO_3_ (Abbatt et al., 2012; Platt & Hönninger, 2003; Pratt et al., 2013; Simpson et al., 2015; Simpson, von Glasow et al., 2007). Although the link between increased bromine in the atmosphere and ozone depletion events was discovered over three decades ago (Barrie, 1986), developing predictive model descriptions of bromine emissions and chemistry in polar regions remains a challenge (Falk & Sinnhuber, 2018; Fernandez et al., 2019; Herrmann et al., 2021; Huang et al., 2020; Toyota et al., 2011; Yang et al., 2008). At present, most models used to predict Arctic scale or global ozone largely ignore or only include simplified descriptions of these processes and do not correctly predict boundary layer ozone concentrations during the Arctic spring (see for example Monks et al., 2015; Emmons et al., 2015). Since ozone is a key atmospheric oxidant and plays a role in virtually all other atmospheric oxidant cycles (e.g., HO_x_ = OH + HO_2_) and acts as a greenhouse gas, inaccurate predictions of Arctic ozone severely limit our ability to understand past and future polar atmospheric chemistry. In addition, the key link between ozone, halogens, and sea ice/snow cover is essential in order to predict future polar conditions and interpret past ice core records and sea ice conditions (Spolaor et al., 2013, 2016). Finally, atmospheric bromine also cause mercury oxidation in the Arctic boundary layer, leading to atmospheric mercury depletion events (AMDEs) and deposition to the cryosphere and ecosystems. Better predicting Arctic mercury oxidation, and human exposure therefore also requires more realistic representation of Arctic halogen chemistry.

The key emitted species that triggers bromine explosion events, ODEs and AMDEs is molecular bromine (Br_2_), which is photolyzed (R1) to form bromine atoms (Br) that quickly react with ozone (R2). This forms another key gas phase species in the reactive bromine/ozone cycle, bromine monoxide (BrO), which can react with HO_2_ to form HOBr (R3). HOBr then photolyzes (R4) to form OH and Br, which has two main impacts. First, the Br radical goes on to further propagate the ozone destruction cycle. Second, the net effect of both (R3) and (R4) is that one HO_2_ radical is converted to the more reactive OH radical. This can increase the amount of OH relative to HO_2_ present during bromine activation, potentially increasing the oxidation rate of chemical species (e.g., volatile organic gases) within the ABL. BrO also undergoes self reaction to reform Br_2_ (R5), which is the dominant Br_2_ formation pathway under sufficiently high BrO concentrations. The resulting effect of equations R1 to R5 is rapid ozone loss, causing ODEs.

(R1) $${Br}_{2}\;\overset{h\nu }{\to }\;2Br$$

(R2) $$Br+O_{3}\to BrO+O_{2}$$

(R3) $$BrO+{HO}_{2}\to HOBr+O_{2}$$

(R4) $$HOBr\;\overset{h\nu }{\to }\;OH+Br$$

(R5) $$BrO+BrO\to {Br}_{2}+O_{2}$$

The source of atmospheric bromine in the Arctic is undoubtedly bromide (Br^−^) that is present in trace amounts in the ocean, and is activated via heterogeneous reactions on surfaces (snow, aerosols, etc). Recycling of reactive bromine via gas phase and heterogeneous reactions on surfaces is crucial in sustaining significant concentrations of atmospheric bromine that cause ODEs. Without this recycling, the quantity of reactive bromine (present in pptv levels) in the atmosphere is too small to sufficiently deplete ozone (present in ppbv levels). Recycling of bromine on surfaces can occur via reactions involving HOBr (R6) and BrONO_2_ (R7) on salty surfaces, resulting in re‐release of Br_2_ to the atmosphere. These heterogeneous processes are what make bromine species incredibly active during polar spring and capable of depleting ozone to near‐zero values. Reactive bromine cycling is terminated when reactive bromine is deactivated upon formation of species that do not undergo gas phase photochemistry or that are inefficient at reforming reactive bromine via heterogeneous reactions (e.g., HBr).

(R6) $$HOBr+{Br}^{-}+H^{+}\overset{(surface)\;}{\to }{Br}_{2}+H_{2}O$$

(R7) $${BrONO}_{2}+{Br}^{-}\overset{(surface)\;}{\to }{Br}_{2}+{NO}_{3}^{-}$$

While numerous theories have been discussed as to how bromine is released to the atmosphere, two main mechanisms, both relying on salty snow, have been tested in 3D numerical models. The first mechanism has proposed that activation of bromine occurs via reactions on surface snow present on sea ice, followed by further recycling of bromine on land and sea ice based snowpacks (Toyota et al., 2011). It also involves heterogeneous recycling on aerosols present within the atmosphere to sustain halogen activation. This mechanism has been tested in the 3D models GEM‐AQ and EMAC (Falk & Sinnhuber, 2018; Toyota et al., 2011, 2014), and very recently WRF‐Chem (Herrmann et al., 2021). There is experimental evidence for this surface snow mechanism: Pratt et al. (2013) reported the photo‐chemical production of molecular bromine from surface snow using chemical ionization mass spectroscopy (CIMS) based on Arctic snow chamber experiments. It has also been shown that bromine activation correlates with the occurrence of first‐year sea ice (Bougoudis et al., 2020; Simpson, Carlson, et al., 2007), and that it can also occur over snow found on multi‐year sea ice (Peterson et al., 2019). These surfaces are similar in that both first year and multi year ice are usually snow covered.

The second mechanism that has been proposed is that bromine activation occurs on aerosols originating from sublimation of salty blowing snow. Under high wind conditions, snow is lofted into the atmosphere and undergoes sublimation to form new sea salt particles in the atmosphere. Fresh sea salt aerosols (primarily sodium chloride, NaCl) contain trace amounts of bromide that undergo heterogeneous chemistry to release reactive bromine to the atmosphere (Huang & Jaeglé, 2017; Yang et al., 2019, 2008, 2010), which is fastest in the presence of sunlight (i.e., photo‐chemical reactions are occurring). There is recent direct evidence for the role of blowing snow in forming sea salt aerosols in the Antarctic (M. Frey et al., 2020). Model studies on polar aerosols also demonstrate an improved agreement compared to sea salt observations for winter and spring when blowing snow sourced sea salt aerosols are included (Huang et al., 2018; Rhodes et al., 2017). Further, this has been recently shown to improve model predictions of BrO and O_3_ (Huang et al., 2020; Yang et al., 2020). Finally, observations show that aerosols can sustain bromine activation above the boundary layer (Peterson et al., 2017), but it has not yet been clearly demonstrated from measurements that blowing snow sourced sea salt aerosols trigger bromine explosion events.

Bromine chemistry is influenced by numerous polar processes including: light availability (influenced by cloud cover, latitude, and season), atmospheric boundary layer dynamics, mixing between the free troposphere and ABL, occurrence of high winds/storms, and other factors (e.g., stratospheric influences). There is a delicate interplay between atmospheric dynamics, emissions, recycling and chemistry, which determines when bromine activation results in significant observable impacts on atmospheric chemistry (Jones et al., 2009; Peterson et al., 2015). For example, the very stable atmospheric boundary layers often found over ice/snow correspond to slow vertical mixing/dispersion and low wind speeds (e.g., Anderson & Neff, 2008). These conditions likely favor the importance of surface emissions from snow on sea ice by concentrating these emissions into a small volume, allowing for the bromine explosion cycle to take off (e.g., Swanson et al., 2020). Conversely, high wind conditions that are found during storms favor blowing snow and blowing snow sourced aerosol formation. Blowing snow sourced aerosols are also likely to be most important when the ocean is mostly ice covered, suppressing open ocean sea salt aerosol production (Huang et al., 2018). High winds also indicate that the ABL is not clearly separated from the free troposphere, allowing air masses containing high bromine to be lofted from the surface to higher altitudes where they can be more easily detected above clouds via satellite remote sensing (Blechschmidt et al., 2016). These complex factors, must be taken into account when considering Arctic halogen chemistry within different 3D modeling frameworks and model evaluations using observations. In this study, we focus on very near surface processes and model evaluation using near surface observations within the ABL.

In this work, we implement a bromine and chlorine chemistry mechanism in an advanced regional meteorological and atmospheric chemistry model, the Weather Research and Forecasting model coupled with Chemistry (WRF‐Chem), to study springtime ODEs in the Arctic in 2012. We include, for the first time two different halogen activation and recycling mechanisms and we study their individual contributions to Arctic ozone depletion events for one example season, spring 2012. Section 2 describes the model setup and an optimized meteorological setup to simulate Arctic boundary layer dynamics and mixing. Section 3 describes the new model developments implemented in WRF‐Chem 4.1.1. In Section 4, we evaluate the performance of these developments by comparing model results with surface measurements of ozone and BrO taken at multiple Arctic sites. In Section 5 we use these new developments to investigate what triggers Arctic ozone depletion events, and to better understand their impacts on Arctic atmospheric chemistry. We put our results and spring 2012 into the long term meteorological and ozone depletion context in Section 6. Finally, the lessons learned are discussed in Section 7.

## 2 Methodology

### 2.1 WRF‐Chem Model Setup

In order to reproduce observed ozone depletion events in the Arctic, we add bromine and chlorine chemistry in the WRF‐Chem 4.1.1 model (Fast et al., 2006; Grell et al., 2005). We perform these developments in a version of WRF‐Chem already optimized for Arctic aerosols and ozone (Marelle et al., 2017), but that to date did not include a description of halogen chemistry. New developments are integrated to the SAPRC‐99 gas‐phase chemistry scheme (Carter, 2000), coupled with the MOSAIC‐8bin sectional aerosol scheme (Zaveri et al., 2008) within WRF‐Chen 4.1.1, due to its skill at reproducing boundary layer aerosols and ozone (outside of ozone depletion events). MOSAIC includes secondary organic aerosols (SOA), aqueous chemistry, and already includes chlorine aerosol species including heterogeneous ClNO_2_ formation from N_2_O_5_.

**Figure 1:**
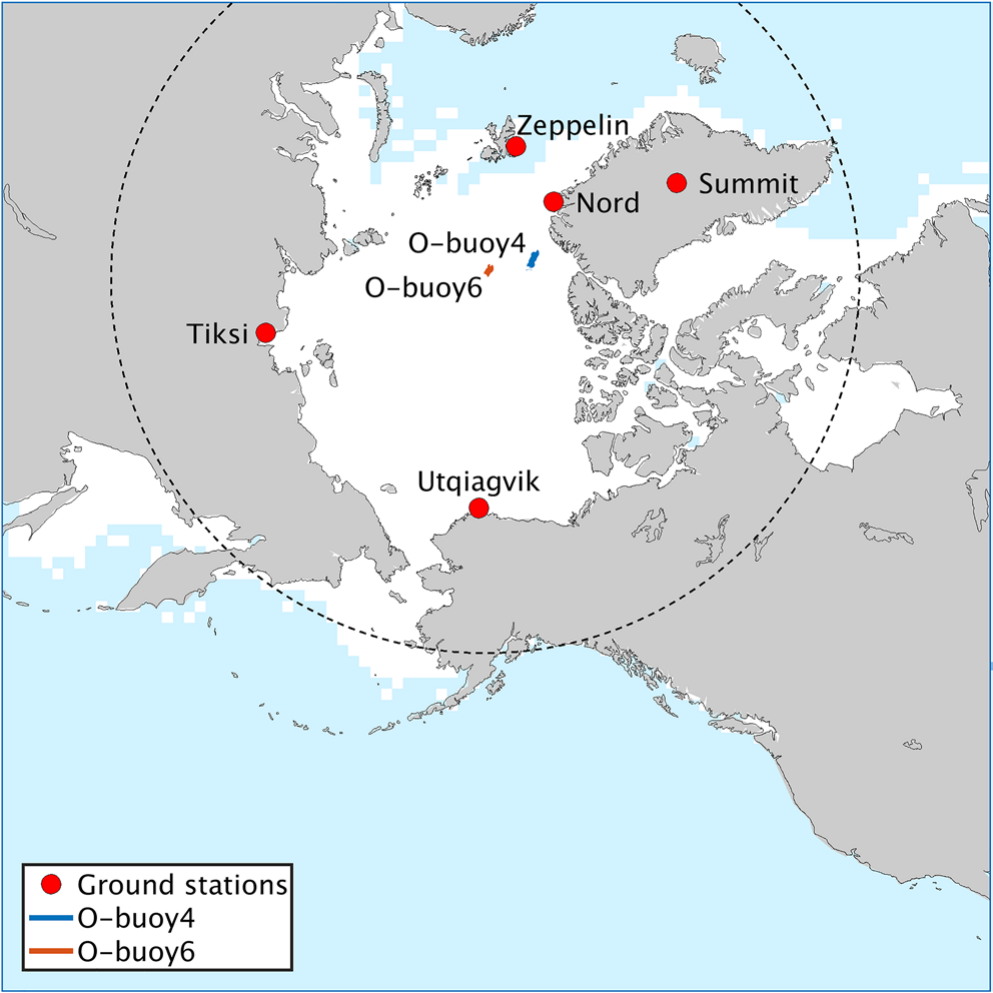
Simulation domain, sea ice cover at the beginning of the simulation, location of the measurement sites, and 60° north latitude circle.

Photolysis rates are calculated by the Fast‐*J* scheme (Wild et al., 2000). Cloud microphysics are represented by the Morrison 2‐moment scheme (Morrison et al., 2009), and cumuli by the KF‐CuP scheme (Berg et al., 2015), which are both coupled to MOSAIC aerosols (wet removal, cloud chemistry, tracer transport, aerosol activation). Longwave and shortwave radiation calculations are performed in the RRTMG scheme (Iacono et al., 2008). Initial and boundary conditions for aerosols and trace gases are from the Model for Ozone and Related chemical Tracers, version 4 (MOZART‐4, Emmons et al., 2010). We chose a model domain centered over the Arctic (domain shown in Figure 1) with a horizontal resolution of 100 × 100 km to encompass the entire Arctic, and a vertical resolution of 72 levels up to a pressure of 50 hPa. All simulations are performed between the dates March 1, 2012 and April 31, 2012. The first 7 days are model spin up and are excluded from the analysis.

### 2.2 Optimized Meteorological Setup for Accurate Boundary Layer Dynamics

An accurate representation of boundary layer dynamics, especially boundary layer stability, is particularly critical for vertical mixing and non‐linear atmospheric chemistry within the ABL. For this reason, we tested and evaluated multiple WRF dynamics configurations in order to select the meteorological options and the global model driving initial and boundary conditions. We tested two different global meteorological datasets ERA‐Interim (Dee et al., 2011) and NCEP FNL (Final Analysis, National Centers for Environmental Prediction, 2000). We also tested three different land surface models: the Noah Land Surface Model (NoahLSM, Tewari et al., 2004), the Noah land surface model with MultiParameterization options (NoahMP, Niu et al., 2011), and the Community Land Model version 4 (CLM4, Oleson et al., 2010). In addition, we tested three different boundary layer schemes: the Yonsei University Scheme (YSU, Hong et al., 2006), the Mellor–Yamada–Janjic Scheme (MYJ, Janjić, 1994), and the Mellor–Yamada Nakanishi Niino Level 2.5 Scheme (MYNN2, Nakanishi & Niino, 2009).

Because not all combinations of options were compatible with each other and with the chemistry and aerosol schemes, in total 13 simulations were completed (Table 1). Simulated 2‐m temperatures from these 13 runs are compared to observations at Utqiaġvik, Alaska (NOAA/ESRL/GMD Baseline Observatories, https://www.esrl.noaa.gov/gmd/dv/data/). In addition, modeled vertical temperature profiles at Utqiaġvik, Alaska are compared to measurements from the Integrated Global Radiosonde Archive (IGRA, Durre et al., 2006), provided twice a day at 11 UTC and 23 UTC. The model is evaluated using radiosondes below 500 meters (altitude above ground level), to evaluate the structure of the lowest portion of the troposphere where halogen chemistry is active. Table 1 shows the root‐mean‐square errors (RMSEs) and correlations between each chosen setup and these two observational datasets.

**Table 1:** Root Mean Square Error (RMSE) and Correlation Coefficient (R) Between WRF Simulations and Temperature Observations (IGRA Radiosondes, Surface Measurements From NOAA) at Utqiaġvik, Alaska

Driving model	Surface	Boudary layer	R_IGRA_	RMSE_IGRA_(K)	R_surface_	RMSE_surface_(K)
ERA‐Interim	CLM	YSU	0.44	2.21	0.94	4.34
ERA‐Interim	CLM	MYNN2	0.48	2.59	0.94	4.37
ERA‐Interim	Noah‐MP	YSU	0.47	2.27	0.94	4.00
ERA‐Interim	Noah‐MP	MYJ	0.49	2.55	0.94	4.23
ERA‐Interim	Noah‐MP	MYNN2	0.48	2.55	0.94	4.09
ERA‐Interim	Noah LSM	YSU	0.49	2.57	0.94	3.94
ERA‐Interim	Noah LSM	MYJ	0.49	2.16	0.95	3.84
ERA‐Interim	Noah LSM	MYNN2	0.46	2.60	0.95	3.92
FNL	CLM	YSU	0.46	2.23	0.93	4.13
FNL	CLM	MYNN2	0.45	2.60	0.93	4.20
FNL	Noah LSM	YSU	0.47	2.23	0.94	3.77
FNL	Noah LSM	MYJ	0.51	2.58	0.94	3.76
**FNL**	**Noah LSM**	**MYNN2**	**0.53**	**2.53**	**0.94**	**3.79**

*Note*. Selected model setup for this study is shown in bold.

**Figure 2:**
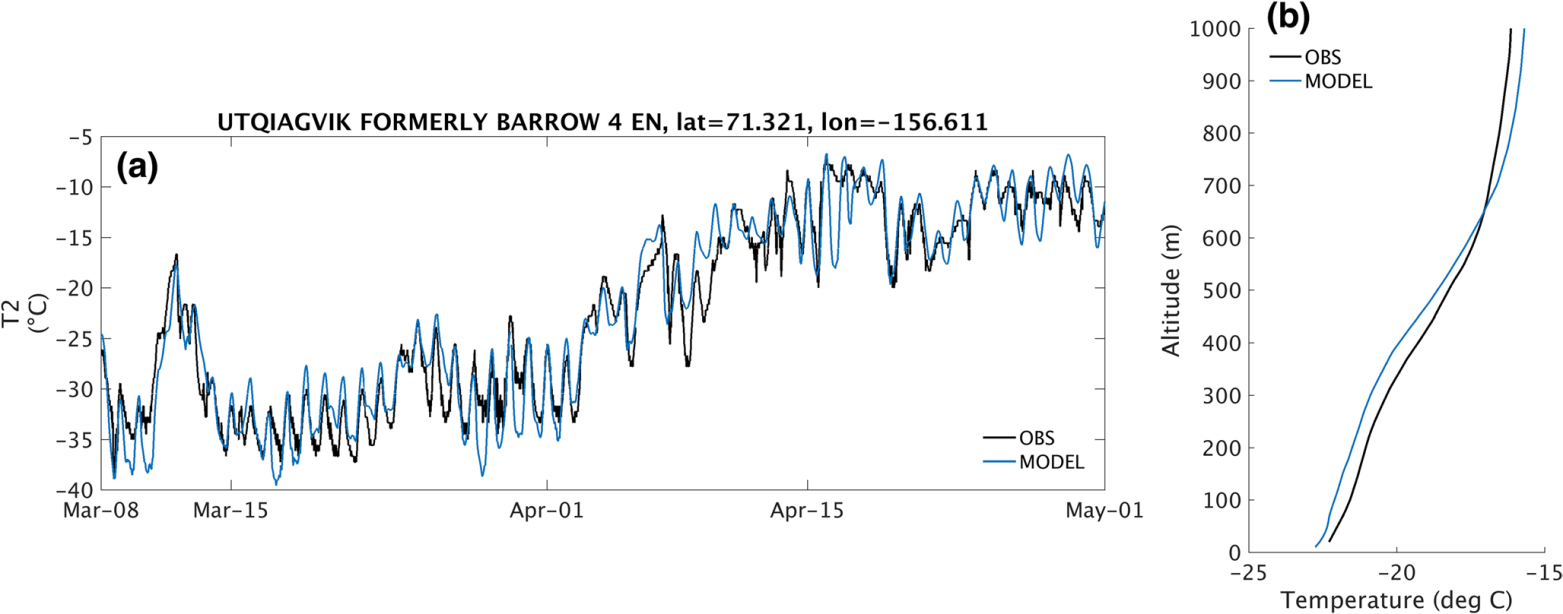
(a) 2‐m temperature observed (black) and simulated by our selected WRF setup (blue) at Utqiaġvik, Alaska. (b) Average temperature profile observed by radiosondes over Utqiaġvik (black) during the same period, and interpolated (land points only) at the same locations and times in our selected WRF setup (blue).

Since boundary layer structure is critical in capturing dispersion and chemistry of surface emissions, we chose the setup with the highest correlation with IGRA radiosonde measurements. This good agreement is illustrated in Figure 2. This setup uses the FNL (final) analysis from NCEP for initial conditions, boundary conditions and spectral nudging; the Noah Land Surface Model; and the MYNN2 boundary layer scheme with the MYNN2 surface layer. This setup also performs well against other metrics. Figure 2 also illustrates that even though it does not have the best agreement with 2‐m temperatures at Utqiaġvik, the model performance there is still very good. The following model runs are therefore all performed with this model setup.

## 3 New Model Developments in WRF‐Chem 4.1.1

We have added to the model chlorine and bromine gas phase reactions including photolysis (Section 3.1), heterogeneous halogen reactions on aerosols (Section 3.2), dry and wet deposition of halogen species (Section 3.3), and emissions of bromine from sea‐ice, snow, and open oceans (Section 3.4). The version of the model used in this study and the input files are publicly available as Marelle et al. (2021).

### 3.1 Gas‐Phase Chlorine and Bromine Chemistry

We add 82 additional gas‐phase chemical reactions involving chlorine and bromine, and 50 additional gas‐phase species, to the Kinetic PreProcessor (KPP) within WRF‐Chem. These reactions are taken from a combination of prior modeling work on chlorine and bromine chemistry (Gratz et al., 2015; Piot & Glasow, 2009; Thomas, Dibb, Huey et al., 2012; Thomas et al., 2011; von Glasow et al., 2002a, 2002b).

These 82 new gas‐phase reactions include 15 photolysis reactions. The new photolysis rates are calculated in the Fast‐J photolysis scheme in WRF‐Chem (Wild et al., 2000), using absorption cross sections and quantum yields from IUPAC (Atkinson et al., 2008, http://iupac.pole-ether.fr/). Cross‐sections and yields are taken from NASA JPL instead (Burkholder et al., 2015) when species were not found in IUPAC (BrO, OClO), or when JPL data were more spectrally resolved or covered a larger spectral range (Br_2_, BrNO_2_). In order to be used in Fast‐J, the JPL and IUPAC cross sections at high spectral resolution are weighted by the solar spectrum and distributed to the seven coarse Fast‐J spectral bins. The preprocessor used to perform this interpolation (Wild et al., 2000) is available along with input files at https://github.com/lmarelle/FastJ-preprocessor.

### 3.2 Heterogeneous Reactions Involving Halogens

Heterogeneous reactions on aerosols containing bromine or chlorine are an important step sustaining activation of gaseous halogen species in the Arctic. We include a parameterized representation of halogen heterogeneous chemistry in the SAPRC‐99 MOSAIC‐8bin scheme within WRF‐Chem, for the 12 heterogeneous reactions presented in Table 2. Following Dentener and Crutzen (1993), we assume that the rate limiting factor in these heterogeneous reactions is the uptake of gaseous species on the aerosol. The heterogeneous reactive uptake coefficient *γ* is corrected by the unitless factor, *J*, which is dependant on *γ* and the Knudsen number (*Kn*). *J*_*n*_ represents the limitation of reaction at the aerosol surface due to gas diffusion limitations, which is calculated for each aerosol size bin following Equation 1 in Fuchs and Sutugin (1971), as presented in Seinfeld and Pandis (1998).

(1) $$J_{n}=\frac{0.75\gamma \left(1+Kn\right)}{Kn^{2}+Kn+0.283Kn\times \gamma +0.75\gamma }$$

**Table 2:** Heterogeneous Reactions, Reaction Probabilities (*γ*) and Yields (*ϕ*)

Reaction	Representation in WRF‐Chem	*γ* and *ϕ*	Ref.
$HOCl\overset{aerosol}{\to }{Cl}_{2}$	HOCl(+HCl) → Cl_2_	*γ* = 0.0004, *ϕ* = 0.5	a
$HOCl\overset{aerosol}{\to }BrCl$	HOCl(+HBr) → BrCl	*γ* = 0.0004, *ϕ* = 0.5	a
${ClONO}_{2}\overset{aerosol}{\to }{Cl}_{2}+{HNO}_{3}$	ClONO_2_(+HCl) → Cl_2_ + HNO_3_	*γ* = 0.11, *ϕ* = 0.27	b
${ClONO}_{2}\overset{aerosol}{\to }BrCl+{HNO}_{3}$	ClONO_2_(+HBr) → BrCl + HNO_3_	*γ* = 0.11, *ϕ* = 0.46	b
${ClONO}_{2}\overset{aerosol}{\to }HOCl+{HNO}_{3}$	ClONO_2_ → HOCl + HNO_3_	*γ* = 0.11, *ϕ* = 0.27	b
$HOBr\overset{aerosol}{\to }{Br}_{2}$	HOBr(+HBr) → Br_2_	*γ* = 0.1, *ϕ* = 0.5	c, d
$HOBr\overset{aerosol}{\to }BrCl$	HOBr(+HCl) → BrCl	*γ* = 0.1, *ϕ* = 0.5	c, d
${BrONO}_{2}\overset{aerosol}{\to }Br2$	BrONO_2_(+HBr) → Br_2_	*γ* = 0.14, *ϕ* = 0.42	e
${BrONO}_{2}\overset{aerosol}{\to }BrCl$	BrONO_2_(+HCl) → BrCl	*γ* = 0.14, *ϕ* = 0.29	e
${BrONO}_{2}\overset{aerosol}{\to }HOBr+{HNO}_{3}$	BrONO_2_ → HOBr + HNO_3_	*γ* = 0.14, *ϕ* = 0.29	e
$N_{2}O_{5}\overset{aerosol}{\to }{BrNO}_{2}+{HNO}_{3}$	N_2_O5(+HBr) → BrNO_2_ + HNO_3_	*γ* = 0.044, *ϕ* = 0.24	f
$OH\overset{aerosol}{\to }{Cl}_{2}$	OH(+HCl) → 0.5*Cl_2_	*γ* = 0.2, *ϕ* = 0.5	g, h

^a^ Ammann et al. (2013).

^b^ Aguzzi and Rossi (1999).

^c^ Pratte and Rossi (2006).

^d^ International Union of Pure and Applied Chemistry (2009).

^e^ Deiber et al. (2004).

^f^ Seisel et al. (1998).

^g^ Knipping et al. (2000).

^h^ Laskin et al. (2006).

We model the heterogeneous reaction rate *k* (s^−1^) following Equation 2 as a product of *J*, the total aerosol area density *A* (cm^−1^), the mean molecular speed ($\bar{v}$, given in cm s^−1^), *γ*, and the yield (*ϕ*) representing the weight of the different possible reaction pathways for a given species on the aerosol (such that the sum of the different reaction yields for a given species is 1). The *γ* values for each reaction and yields used are given in Table 2. Reaction rates are calculated for each of the eight MOSAIC aerosol size bins and then summed to obtain the total heterogeneous reaction rate.

(2) $$k=\sum_{i=1}^{n=8}0.25\phi \bar{v}\gamma A_{i}J_{i}$$

This approach allows us to represent the effect of heterogeneous chemistry on the gas phase, without explicitly calculating the full chemistry in the aerosol phase. In order to limit the computational cost of the new scheme, we do not model aerosol bromine explicitly either, since adding an additional aerosol species in the MOSAIC‐8bin aerosol scheme adds 16 advected tracers to the scheme (eight interstitial aerosol bins and eight cloud‐borne). In order to lighten the mechanism and still maintain mass conservation for bromine, reactions consuming aerosol‐phase bromine (e.g., HOCl $\overset{(aerosol)\;}{\to }$ BrCl) are rewritten using HBr as a proxy for aerosol bromine (e.g., HOCl + HBr → BrCl) (Table 2). For each of these reactions, the heterogeneous reaction rate is divided by the HBr concentration in KPP, to keep the kinetics independent of the HBr concentration while still consuming HBr (following Badia et al., 2019)

For consistency and to simplify model developments, we use the same approach, using HCl, for heterogeneous reactions consuming chlorine in aerosols, even though chlorine aerosols are represented explicitly in MOSAIC‐8bin. The only exception is the heterogeneous formation of ClNO_2_ through N_2_O_5_, which is already calculated explicitly in MOSAIC in WRF‐Chem 4.1.1.

Some of these heterogeneous reactions might require acidic conditions to proceed (Abbatt et al., 2012). For aerosols that have a pH calculated in MOSAIC, we chose an aerosol pH threshold of five, above which the heterogeneous reaction rates (Equation 1) are set to 0. This pH condition is checked for each aerosol size bin independently, before calculating the summed reaction rates for the full aerosol population in Equation 2.

### 3.3 Dry and Wet Deposition of Halogen Species

We include dry deposition for seven new halogen species: Br_2_, HOBr, HBr, BrONO_2_, Cl_2_, HOCl and ClONO_2_. Dry deposition is neglected for all other new species. Dry deposition is calculated through the resistance scheme of Wesely (1989). This scheme requires four parameters for each new species: the effective Henry's law constant (H*); the Henry's law temperature correction factor (DHR); the deposition reactivity parameter (f0, representing the reactivity of the species when in contact with the ground surface); and the molecular diffusivity of the species (dvj). The values of these variables for the seven new species are presented in Table 3. H* and DHR are taken from Sander (2015), f0 from Toyota et al. (2011), and dvj is taken as the inverse square root of the species molecular weight in g mol^−1^.

**Table 3:** Parameters for the Dry Deposition Scheme: Henry's Law Constant (H*), Henry's Law Temperature Correction Factor (DHR), Deposition Reactivity Parameter (f0), Molecular Diffusivity (dvj)

Species	H*(mol m^−3^ hPa^−1^)	DHR(K)	f0	dvj(cm^−^2 s^−1^)
Br_2_	0.730	4,400	1	0.079
HOBr	6,000	0	1	0.102
HBr	24.3	370	0	0.111
BrONO_2_	24.3	370	1	0.084
Cl_2_	0.0932	2,000	1	0.12
HOCl	659	5,900	1	0.14
ClONO_2_	1,510	2,300	1	0.1

Wet removal of HCl was already included in WRF‐Chem 4.1.1. We added to the model wet deposition of HBr, HOBr, BrONO_2_, HOCl and ClONO_2_ by impaction scavenging, using a first‐order scavenging rate constant of 3.89 × 10^−4^ s^−1^ per mm h^−1^ of precipitation (Toyota et al., 2011).

### 3.4 Emissions of Bromine From Sea‐Ice, Snow, and Open Oceans

Emissions of bromine in the Arctic have been attributed to multiple sources including sea‐ice (first‐year and multi‐year), snow surfaces, sea salt from blowing snow, and oceanic sea salt. We implemented descriptions of these emission sources in WRF‐Chem 4.1.1, which are described in the following sections.

#### 3.4.1 Br_2_ Emissions From Surface Snow

Surface snow bromine activation follows Toyota et al. (2011). In this mechanism, deposition of atmospheric oxidants to the snowpack over sea ice releases Br_2_ to the atmosphere, and this process is photochemically accelerated in the presence of sunlight. In practice, the Br_2_ emission flux is calculated as proportional to the O_3_ dry deposition flux, with a proportionality factor depending on solar zenith angle. In sunlit conditions (solar zenith angle ≤ 85°), Br_2_ emissions are 0.075 times the deposition flux, and in dark conditions, 0.001 times.

#### 3.4.2 Br_2_ Emissions From Blowing Snow

The blowing snow parameterization is based on Yang et al. (2008) and Huang and Jaeglé (2017). Blowing snow events start when the 10‐m wind speed, *w*10, is above the threshold *w*10_crit_, which is a function of surface temperature (Yang et al., 2008). Lofted snow sublimates in the atmosphere depending on environmental conditions, releasing sea salt aerosols and Br_2_. In WRF‐Chem 4.1.1, we calculate the Br_2_ emission flux, $E_{Br_{2}}$ (kg m^−2^ *s* ^−1^), following Equation 3.

(3) $$E_{Br_{2}}=\sum_{i=1}^{n=8}E_{NaCl}\left(bin\right)\times R_{a}\times DF$$

where *E*_NaCl_(bin) is the sea salt emission flux from blowing snow in a given MOSAIC aerosol size bin (kg m^−2^ *s* ^−1^), *R*_*a*_ is the mass ratio between bromine and NaCl in sea water (0.00233), and DF is the maximum bromine depletion factor of 0.38 from Yang et al. (2008), based on Sander et al. (2003), representing the fraction of aerosol bromine lost to the atmosphere. This maximum value for the depletion factor represents an estimate of bromine emissions emitted during the whole atmospheric lifetime of the blowing‐snow sourced sea salt aerosols. This constant value was chosen to limit the computational cost of the new scheme, since in reality bromine emissions from sea salt aerosols depend on heterogeneous chemistry on the aerosols, which can only be resolved by explicitly tracking the simulated size‐resolved aerosol bromine chemistry and resulting aerosol bromide content.

The sea salt emissions in each MOSAIC aerosol size bin, *E*_Nacl_(bin), are calculated following Equation 4.

(4) $$E_{Nacl}\left(bin\right)=\frac{q_{s}\xi }{1000}\int_{D_{low}\left(bin\right)}^{D_{high}\left(bin\right)}f\left(D_{dry}\right)dD_{dry}$$

where *q*_*s*_ is the snow sublimation flux (kg m^−2^ *s* ^−1^), calculated as a function of local wind speed, temperature and humidity (Yang et al., 2008). In Equation 4, *D*_high_ (bin) and *D*_low_ (bin) are also the lower and upper dry diameter range of a given MOSAIC aerosol size bin, *f* (*D*_dry_) is the snow size distribution expressed as a function of dry sea salt aerosol size (Yang et al., 2008), and *ξ* is a uniform snow salinity of 0.1 psu (Huang & Jaeglé, 2017). Following Huang and Jaeglé (2017), we also assume that each snow flake emits *N* = 5 sea salt aerosols.

Available values of salinity from the Antarctic differ by more than an order of magnitude (Rhodes et al., 2017). The Massom et al. (2001) distribution used in Yang et al. (2008) has a mean value of 8.3 psu, 83 times higher than the Arctic value used in our implementation. No pan‐Arctic measurements of snow and snow on sea ice salinity currently exist, but recent measurements in the central Arctic (M. M. Frey & Nomura, 2019) found snow salinities with a median value of 0.02 psu and a mean of 1.7 psu. Here, we use the parameters from Huang and Jaeglé (2017) (*ξ* = 0.1 psu, *N* = 5), which are in the middle of this range, and were shown to improve model agreement with observed sea salt aerosol concentrations at Utqiaġvik. In agreement with Huang and Jaeglé (2017), we show (Supplementary Figure S1) that the chosen values for *ξ* and *N* produce more realistic sea salt aerosol concentrations at Arctic coastal sites than the original Massom et al. (2001) salinity and *N* = 1 value used in Yang et al. (2008). We also show that low salinity values of 0.01 psu match observations even better than 0.1 psu, while a reasonable high bound of 1.7 psu leads to overestimations of Na aerosols by up to two orders of magnitude.

#### 3.4.3 Bromine Recycling on Surface Snow

Br_2_ emitted to the atmosphere by either surface snow or blowing snow can be transformed into HOBr and BrONO_2_. When deposited on sea ice and snow, these species can be recycled back into atmospheric Br_2_ by surface reactions in the snowpack. Following Toyota et al. (2011), we assume that all HOBr and BrONO_2_ deposited on sea ice is re‐emitted as Br_2_. This assumes an unlimited supply of Br^−^ in snow over sea ice. Unlike Toyota et al. (2011), we assume that this recycling is independent of sea ice age, since recent observations indicate that multiyear ice can be an efficient source of Br_2_ (Peterson et al., 2019). Over continental snow, Br^−^ availability in the snowpack is assumed to be limited by HBr deposition. As a result, the Br_2_ emission rate there is limited by the HBr deposition rate, and is equal to the smaller deposition flux between HBr and HOBr + BrONO_2_.

#### 3.4.4 Temperature and Ice Fraction Dependence of Bromine Emissions and Recycling

Recent observations indicate that Br_2_ emissions and recycling can occur at temperatures up to 0°C (Burd et al., 2017). For this reason, we removed the temperature threshold of −15°C used in Toyota et al. (2011) for surface emissions, and replaced it by a 0°C threshold that applies for all Br_2_ emission processes over surface snow (surface snow, blowing snow, surface recycling). When the skin temperature over snow or ice exceeds the 0°C threshold in a grid cell (i.e., when snow starts to melt), the grid cell stops emitting bromine until the end of the simulation.

Snow on sea ice is also influenced by sea ice flooding events, which are more common for thinner and lower fractional sea ice cover (Provost et al., 2017). These events may deactivate snow on sea ice by changing the pH and/or structure of the snow to one less active for bromine release. Due to this, we include a cutoff for halogen activation and recycling on snow on sea ice that is dependent on the grid cell sea ice fraction. We test different fractional sea ice cutoff values (see electronic supplement), which are discussed further in section 4.

#### 3.4.5 Direct Br_2_ Emissions From Open Oceans

Sea salt emitted from open oceans can also release bromine to the atmosphere. We include this source of atmospheric Br_2_ in the model, following Equation 5.

(5) $$E_{Br_{2},ocean}=E_{NaCl,ocean}\times R_{a}\times DF$$

where *E*_NaCl, ocean_ is the sea salt emission flux from the ocean surface (kg m^−2^ *s* ^−1^), already calculated in WRF‐Chem 4.1.1 for ice‐free ocean grid cells (Gong et al., 1997). We added emissions from open leads in sea ice in WRF‐Chem 4.1.1 by calculating the flux for fractional sea ice cells, and scaling it by the open ocean fraction in the cell. *R*_*a*_ is the same Br/NaCl mass ratio already used in the blowing snow parameterization, and *DF* is a mean depletion factor of 0.25.

## 4 Model Results and Evaluation

In order to evaluate the updated model, we performed four different WRF‐Chem simulations, listed in Table 4, and compared them to surface ozone and BrO observations at five different Arctic locations. First, we perform a reference simulation with no halogen chemistry and no updates implemented (NOHALO), and one simulation (BOTH) including all our halogen chemistry developments with both the surface activation mechanism (Section 3.4.1), and the blowing snow parameterization (Section 3.4.2). In order to understand which initial source of atmospheric bromine, (1) surface snow or (2) blowing snow, triggers ozone depletion events in the Arctic, we perform two additional simulations, SURFACE and BLOWING. SURFACE is a simulation with only the surface mechanism included, where blowing snow emissions are excluded. BLOWING is the simulation with only the blowing snow source, where surface emissions presented in Section 3.4.1 are excluded; however we note that the BLOWING simulation still includes bromine recycling on the snow surface (Section 3.4.3), even though it was not included in the original publications of Yang et al. (2008) and Huang and Jaeglé (2017). For the BOTH simulation, we have tested four different fractional sea ice coverage cutoff values for both halogen activation and recycling mechanisms to be active; 15%, 50%, 75%, and 90% (see Section 3.4.4). Based on these tests (Figures S2 and S3 in the electronic supplement), we have chosen a 75% fractional sea ice cover cutoff for all simulations presented.

**Table 4:** Description of the Simulations Performed in This Study

Simulation name	Description
NOHALO	No halogen chemistry and no updates included
SURFACE	Only surface activation mechanism implemented (as described by Toyota et al. (2011))
BLOWING	Only blowing snow parameterization included (as proposed by Yang et al. (2008) using parameters from Huang and Jaeglé (2017))
BOTH	Both SURFACE and BLOWING mechanisms operating

Abbreviations: BOTH, one simulation; NOHALO, simulation with no halogen chemistry and no updates implemented.

### 4.1 Surface Ozone and BrO Evaluation at Utqiaġvik, Alaska

**Figure 3:**
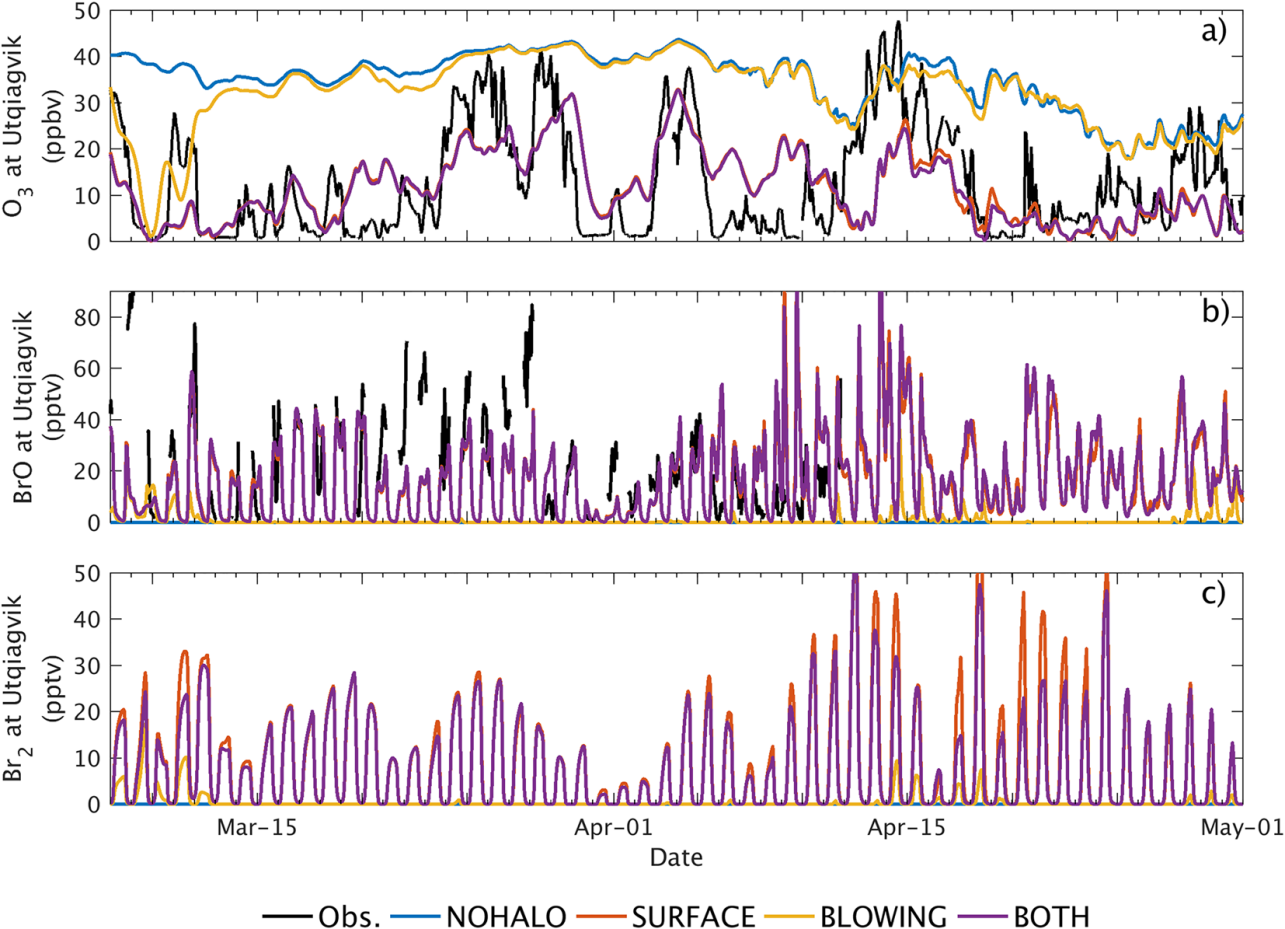
(Top) O_3_ observed (black) at Utqiaġvik, Alaska and simulated by WRF‐Chem in the simulation with no halogen chemistry and no updates implemented (blue), SURFACE (orange), BLOWING (yellow) and BOTH (purple) simulations. (Middle) BrO observed by MAX‐DOAS during the BROMEX campaign at Utqiaġvik, and simulated by WRF‐Chem. (Bottom) Br_2_ simulated by WRF‐Chem at Utqiaġvik.

The comparison between observed and modeled concentrations of O_3_ and BrO at Utqiaġvik, AK (formerly Barrow, AK) is shown in Figure 3. Surface observations of ozone in Utqiaġvik are from NOAA‐ESRL (https://www.esrl.noaa.gov/gmd/dv/data/). BrO is measured by a ground‐based (0–200 m) MAX‐DOAS in Utqiaġvik (BROMEX campaign, described in Simpson et al. [2017]). WRF‐Chem surface Br_2_ concentrations (0–200 m average) are also shown. Model results are spatially interpolated at the location of the measurements, using only land grid cells.

The simulation including both mechanisms (BOTH), captures the observations more accurately than the base version of the model (NOHALO). The RMSE of O_3_ in BOTH is 10 ppbv, compared to 25 ppbv in NOHALO (detailed statistics are given in supplementary Table S1). The variability of ozone is also captured in the model when both emission mechanisms are implemented (correlation coefficient of 0.50 in BOTH, compared to 0.22 in NOHALO). The amount and timing of ozone depletion events are generally well represented, including both large scale ODEs that occur during the simulation period as well as smaller ozone depletion/regeneration events. At this site, surface snow activation (SURFACE simulation) is the main operating mechanism for ozone depletion as it captures most of the large ODEs and smaller peak fluctuations (RMSE 10.3 ppbv, correlation 0.50). The blowing snow mechanism (BLOWING simulation) does influence the modeled ozone levels to a small extent for most of the simulation period, however, it is only able to entirely capture the first ODE of the simulation (starting 8 March 2012) indicating this particular event may be initiated by blowing snow. These developments are able to significantly improve the representation of modeled ODEs, yet reproducing the full nature of all events remains a challenge.

Similarly, Figure 3 shows that the timing of enhanced BrO concentrations reproduced by the model is comparable to the observational data during both periods of increasing and declining BrO concentrations. However, from March 20th to March 30th modeled BrO is underestimated, and from April 8th to April 11th it is overestimated; this may be due to several factors. It is not likely to be caused by measurement error, since Simpson et al. (2017) found that during March 2012, the typical error in BrO measurement was 2–3 pptv. However, the same study found that BrO retrievals were highly correlated at a 30 km scale only as long as sea ice was unbroken. At 100 km resolutions and at a later period in April when leads are more likely to open, it is possible that the WRF‐Chem grid cell averages are less representative of measurements at Utqiaġvik. In addition, bromine activation and recycling is sensitive to boundary layer stability, and even recent reanalysis datasets or advanced regional models such as WRF still struggle to reproduce stable boundary layers over snow (Sterk et al., 2015; C. Wang et al., 2019).

Figure 3 also shows that extended periods of very low O_3_ concentrations are sometimes associated with low concentrations of BrO. Under these conditions of low ozone concentrations, BrO formation is limited by the fact that there is no ozone for Br atoms to react with. In this case, other unobserved species, such as BrNO_*y*_ compounds, may play a role in sustaining bromine chemistry by regenerating Br_2_ (S. Wang et al., 2019). Similar to ozone depletion, the surface snow mechanism plays the most important role in determining enhanced BrO concentrations as well as high modeled Br_2_ mixing ratios at Utqiaġvik, AK.

### 4.2 Surface Ozone Evaluation at Four Additional Arctic Stations and Two Arctic Ocean Buoys

**Figure 4:**
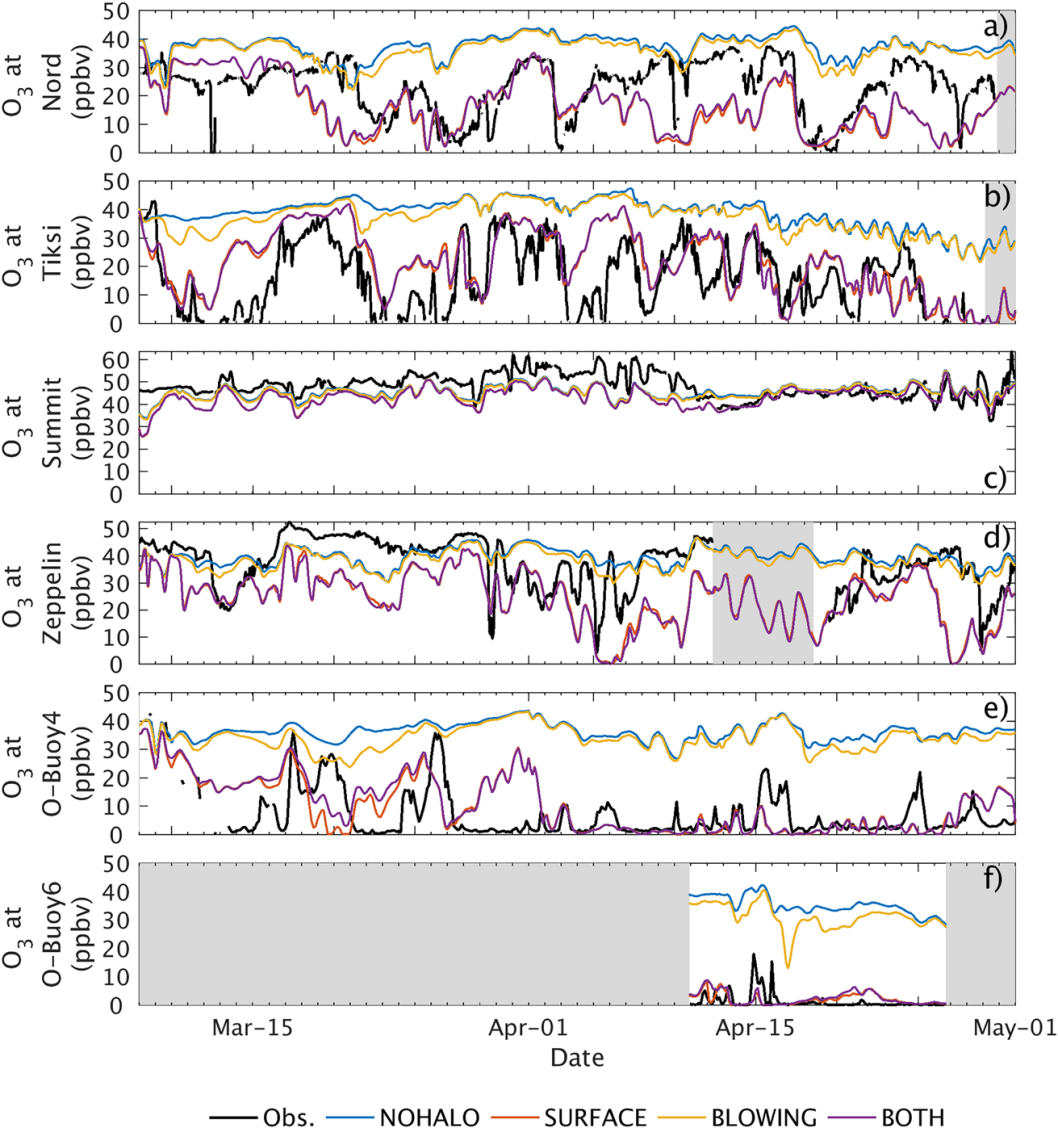
Surface ozone observed (black) and simulated by WRF‐Chem (color) at (a) Station Nord, Greenland; (b) Tiksi, Russia; (c) Summit, Greenland; (d) Zeppelin Station, Svalbard; (e) O‐buoy4, central Arctic; (f) O‐buoy6, central Arctic.

In addition to improvements at Utqiaġvik, we also report improvements in model representation of ozone and ODEs at other Arctic locations. Figure 4 compares the simulated ozone to observations at: Station Nord, Greenland; Tiksi, Russia; Summit, Greenland; Zeppelin Station, Svalbard, and at 2 Arctic buoys in the central Arctic (latitude > 85°N), O‐buoy4 and O‐buoy6 (Halfacre et al., 2014; Knepp et al., 2010; Simpson et al., 2009). Table S1 also gives metrics (RMSE and correlation) at these sites. For high‐altitude sites (Summit and Zeppelin), model O_3_ was interpolated at the altitude of the measurements, even though this altitude was not located in the lowest (surface) model level. At all sites except buoys, spatial interpolation is performed using only land grid cells. Figure 4 (and supplementary Table S1) shows that when the surface scheme or both mechanisms are included, modeled ozone concentrations are greatly improved in Nord, Greenland; Tiksi, Russia, and at buoys in the Central Arctic.

At Station Nord (Figure 4a), a coastal site in the north of Greenland, the base run with no halogen chemistry misses main features of the observed spring ozone mixing ratios in 2012, including ODEs. The BLOWING model simulation has little influence on ozone. We note that we have not tuned the parameters of the surface or blowing snow bromine production mechanism to match observations, so it is possible that this and other events will be better captured upon adjusting the model parameters. As implemented, the surface snow mechanism (SURFACE) captures the timing and features of most events (RMSE = 12.9 ppbv, *R* = 0.33, compared to 17.2 ppbv and 0.17 for NOHALO). At Station Nord, including both mechanisms (BOTH) does not significantly improve the model compared to the surface snow mechanism alone (RMSE = 12.4 ppbv, *R* = 0.34), indicating that blowing snow has limited influence on modeled ozone at this station.

For Tiksi (Figure 4b), a coastal site in Russia, only the surface snow mechanism reproduces the magnitude of observed ODEs. A long ozone depletion event occurs between 9 and 16 March. For this event, the SURFACE simulation predict earlier ozone recovery to background levels. A second ODE, observed in between 22 March and 1 April, is captured by both the SURFACE and BOTH runs, but only very weak ozone depletion occurs in the BLOWING model run. Later, following April 15th, the decay of ozone for an extended period of time is captured by the SURFACE snow model run, but not by the BLOWING simulation. This suggests that in this season, the main operating mechanism is surface snow.

At Summit (Figures 4c and 4a high altitude non‐coastal Arctic site) ozone depletion conditions are not observed. This is due to the high altitude of the site, influenced by free troposphere air masses, and the distance between Summit and the Arctic Ocean. Observations of halogen chemistry at Summit have been completed during other seasons and found 2–3 pptv of bromine can be present during late spring/early summer (Liao et al., 2011; Stutz et al., 2011), however these concentrations are not thought to cause ODEs. In our runs, we have some active bromine chemistry at Summit that arrives via surface snow and aerosol recycling from activation of oceanic bromine sources (Thomas, Dibb, Stutz, et al., 2012). However, the influence of this chemistry is overestimated in our model description and should be investigated further in the future. When using a higher sea ice cover threshold for halogen chemistry of 90% (Supplementary Figure S3), results from BOTH and SURFACE are improved at Summit. Using this higher cutoff disables bromine emissions near Southern Greenland, indicating that some incomplete or missing process in our model (ice flooding, bromine depletion in snow, snow ageing or melt) should disable bromine emissions from these areas. These discrepancies do not persist later in the model run, after April 12th.

At the Zeppelin observatory (Figure 4d, near coastal mountain site) there is no clear signature from blowing snow in modeled ozone depletion. Some of the model‐observation discrepancies for the SURFACE simulation can be explained by the coarse horizontal resolution of 100 km, which is not able to resolve the topography and the local mountain meteorology. Despite these limitations, surface snow does predict the first low ozone event (20 ppbv prior to March 15th), even though the mechanism results in too much ozone depletion. The model then captures a series of ozone depletion events following April 1st, but the BOTH and SURFACE runs remain depleted in ozone while the observations show that ozone recovers quickly. During this period the NOHALO and BLOWING simulation better reproduces the observation. The model cannot be evaluated for several days due to a period of missing measurements centered around April 15th. Then, the model does capture the amount, but not the timing of an ozone depletion event at the end of April. The final event is captured by the SURFACE run.

Observations at very high latitudes at O‐buoy4 and O‐buoy6 indicate that ozone is very often completely depleted in the Central Arctic in Spring 2012. Only the SURFACE simulation (and BOTH) are able to reproduce this very low ozone, although BLOWING reproduces partial depletion around 15–25 March at O‐buoy4, and between 16–18 April at O‐buoy6. Average observed ozone at O‐buoy4 during the whole period is 6.7 ppbv (vs. 8.6 ppbv in SURFACE). O‐buoy6 only has limited data coverage (15 days in late April), and experienced data quality issues (baseline levels are at −1.1 ppbv, corrected on Figure 4f by assuming that the error is a constant offset), but it measured average ozone of 1.3 ppbv during this limited period, also consistent with the 2.0 ppbv average in SURFACE.

In summary, the timing and intensity of the ODEs in the BOTH and SURFACE simulation best captures the overall features within the observations, although the intensities of some events can be either over or underestimated. The average RMSE and correlation of SURFACE against ozone at the seven locations shown in Figures 4 and 3 is 10.2 ppbv and 0.37 respectively, compared to 19.4 ppbv and 0.28 for BLOWING (supplementary Table S1). In addition, we show on supplementary Figure S10 that this is not likely to be due to our choice of parameters for the blowing snow scheme: lower and upper bound snow salinities of 0.01 psu and 1.7 psu still do not reproduce observations as well as SURFACE. The blowing snow simulation with 1.7 psu reproduces observed ozone at Zeppelin relatively well, however supplementary Figure S1 shows that it also produces far too much sea salt at the same site, indicating that bromine emissions by blowing snow are overestimated by the scheme at Zeppelin. In almost all cases, surface snow activation can then be seen as the dominant mechanism for ozone depletion in March–April 2012.

## 5 Origins and Impacts of Springtime Arctic Ozone Depletion

### 5.1 Origin of Ozone Depleted Air Masses at Utqiaġvik

To identify the origin of ozone‐rich and ozone‐depleted air masses, we use the Lagrangian particle dispersion model FLEXPART‐WRF (Brioude et al., 2013), which is a version of the FLEXPART model (Stohl et al., 2005) driven by the WRF meteorological model. Using the meteorological fields from the WRF‐Chem simulation described in Section 2.1, we use FLEXPART‐WRF in backward mode to study the source and transport of ozone‐rich (measured O_3_ > 30 ppb) and ozone‐depleted (measured O_3_ < 10 ppb) air masses during the month of April 2012. For each case, a fixed number of air parcels were released every hour when observed ozone was above or below these thresholds, so that the total number of parcels released from Utqiaġvik, AK was 100,000. For the ozone‐rich air, this represented a total of 68 releases during the month of April, and for the ozone‐depleted air this included 388 releases. Each simulation was run backwards in time for 7 days to track the origin of air measured at Utqiaġvik, AK and to study source‐receptor relationships. To do this, we use surface potential emission sensitivities (PES), calculated by FLEXPART‐WRF, which indicates when air was in contact with the surface and would be sensitive to emissions. PES values are given as the amount of time spent by parcels in each grid cell during the simulation.

**Figure 5:**
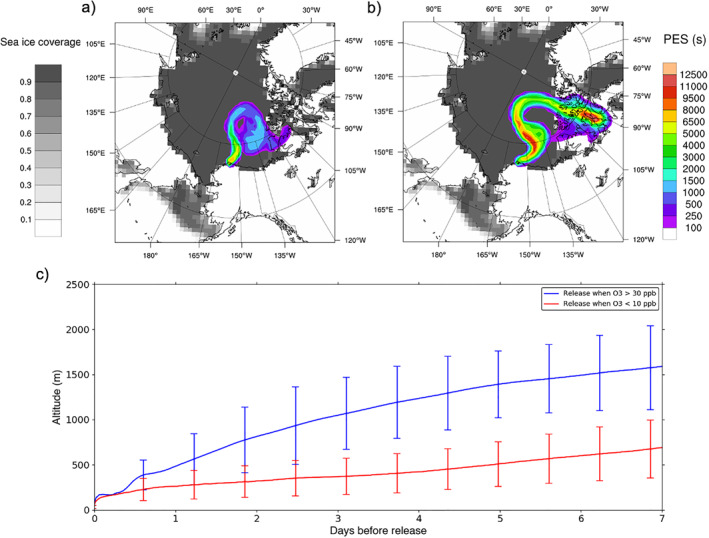
FLEXPART‐WRF 7‐days backward potential emission sensitivity (PES) (a) 0–100 m PES for releases when measured O_3_ was below 10 ppbv and (b) 0–5,000 m PES for when O_3_ exceeded 30 ppbv. Monthly average fractional sea ice coverage, as represented in WRF, for April 2012 is shaded in gray. (c) The altitude (above sea level) of the air mass trajectories, up to 7 days prior to the release, for high background ozone (blue) and low background ozone (red), with RMS error bars.

Figure 5a shows the 0–100 m (surface) PES column, which represents the area where ozone‐depleted air (<10 ppbv) originates from. These air masses originate predominantly from over sea ice for the entire 7 day period prior to measurement. Figure 5b shows the 0–5,000 m PES (consistent with the air altitude in Figure 5c), which shows that high ozone air (>30 ppbv) is subject to long range transport across the Arctic from Siberia during the 7 days prior to arriving at Utqiavik. Figure 5c shows the mean altitude of the transported plumes for low and high ozone air. This shows that during periods of high ozone, the air descends down from the free troposphere prior to measurement at the surface. This downward vertical mixing of ozone rich air from the free troposphere is important for replenishing ozone, and may also allow for new initiation of bromine activation on surface snow. It also allows for generation of BrO in the boundary layer for conditions where bromine is present but BrO is not formed due to complete ozone depletion. This shows there is a complex interplay between triggering at the surface and replenishment of ozone from above the boundary layer, mixed down from aloft. Conversely, during low ozone periods, air remains near the surface, where it is more sensitive to surface and blowing snow emissions and to chemistry occurring on sea salt aerosols within the boundary layer (Figure 5).

### 5.2 Impacts on Pan‐Arctic Surface O_3_, BrO, and HO_x_ During Spring 2012

**Figure 6:**
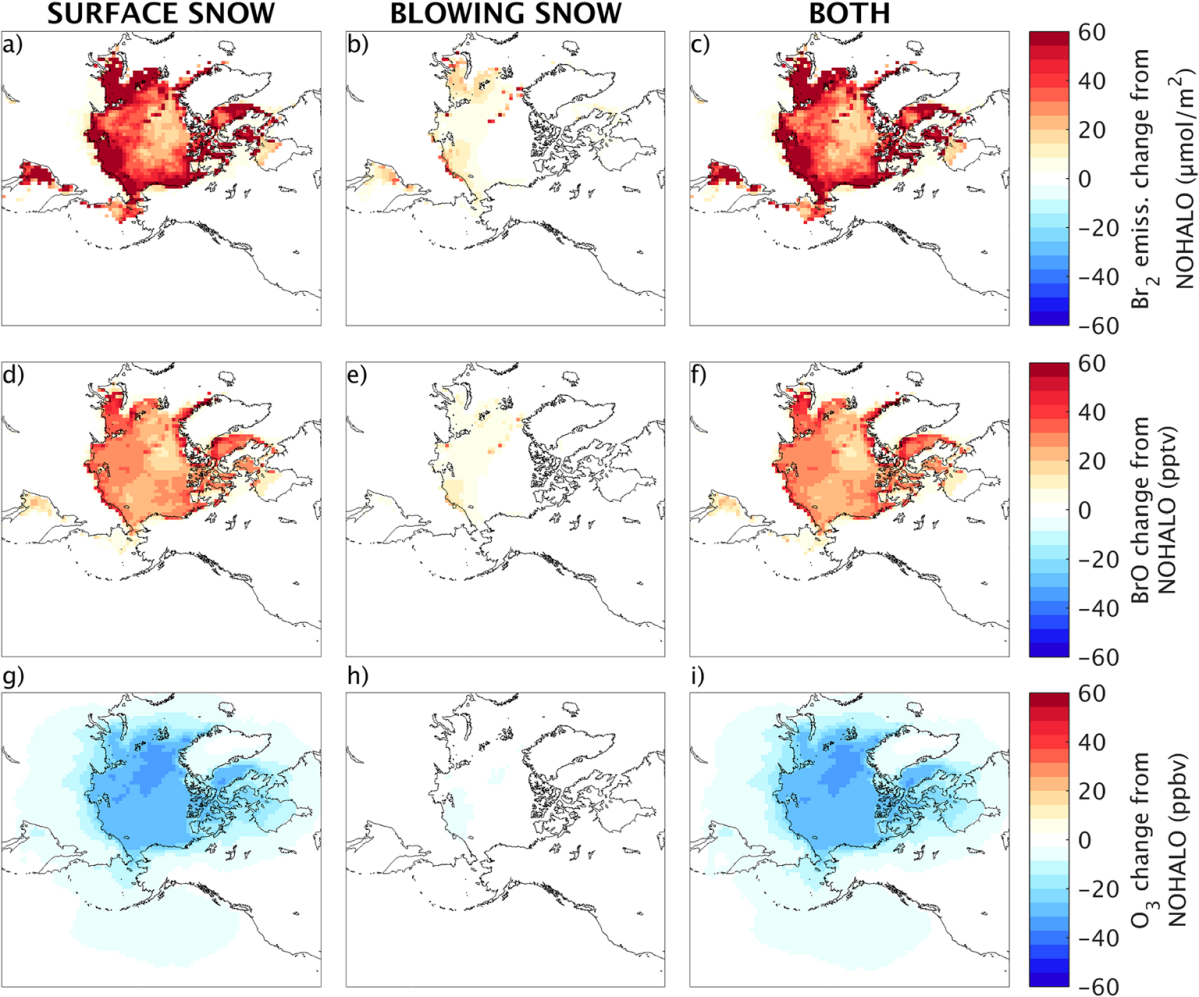
Monthly averaged (April 1–30, 2012) changes in modeled quantities in each simulation, compared to simulation with no halogen chemistry and no updates implemented (NOHALO). Modeled Br_2_ emissions (top), BrO concentrations (middle) and surface ozone concentrations (bottom). Changes predicted across the Arctic for SURFACE‐NOHALO (left panels), BLOWING‐NOHALO (center panels), and BOTH‐NOHALO (right panels).

The independent roles of the two halogen activation mechanisms on surface ozone and BrO concentrations, as well as their effect on Br_2_ emissions, are illustrated in Figure 6. Here, we plot results for the SURFACE (left panels: a, d, g), BLOWING (center panels: b, e, h), and BOTH (right panels: c, f, i) runs, compared to the NOHALO base case, to illustrate how each mechanism activates bromine and impacts ozone.

We plot the total Br_2_ emissions increase from each mechanism in Figures 6a–6c. The most active Br_2_ emissions from surface snow are located on the coastal Arctic. Due to the lack of multi‐year sea ice in 2012 and the recent evidence that bromine is activated from snow on multi‐year sea ice (Peterson et al., 2019), we do not distinguish ice type in the surface snow activation mechanism. This is evident in the emissions from snow on sea ice, which occurs for all sea‐ice covered regions. The key trigger for initial Br_2_ emissions is ozone deposition to sea ice in the surface snow mechanism, therefore emissions may be limited by the lack of ozone deposition when ozone has been depleted to near zero levels in the center of the Arctic (Figure 6g, discussed below, and supplementary Figure S6). For the blowing snow mechanism, the Br_2_ emissions are highest along the Russian coast, Svalbard, and in the Central Arctic, but they are much lower at the Arctic scale. As a result, they contribute relatively little to emissions in the BOTH simulation (Figure 6c), which are dominated by surface snow. We also show in Figures 6d–6f that predicted BrO concentrations do not directly correlate to the Br_2_ emissions locations.

Figures 6g–6i show that, in April 2012, the surface snow mechanism is the main driver for large scale ozone depletion over most parts of the Arctic. This also shows that the effect of blowing snow is much smaller during this month, contributing at most to 10%–20% of total depletion along Eastern Russia (supplementary Figure S7). We also note that ozone depletion and BrO are not well correlated in the central Arctic, where BrO formation is limited by near‐total ozone depletion (mean concentrations ∼5 ppbv, Figure S6). Ozone depletion also extends further inland into the Arctic than bromine activation, as indicated by BrO concentrations.

**Figure 7:**
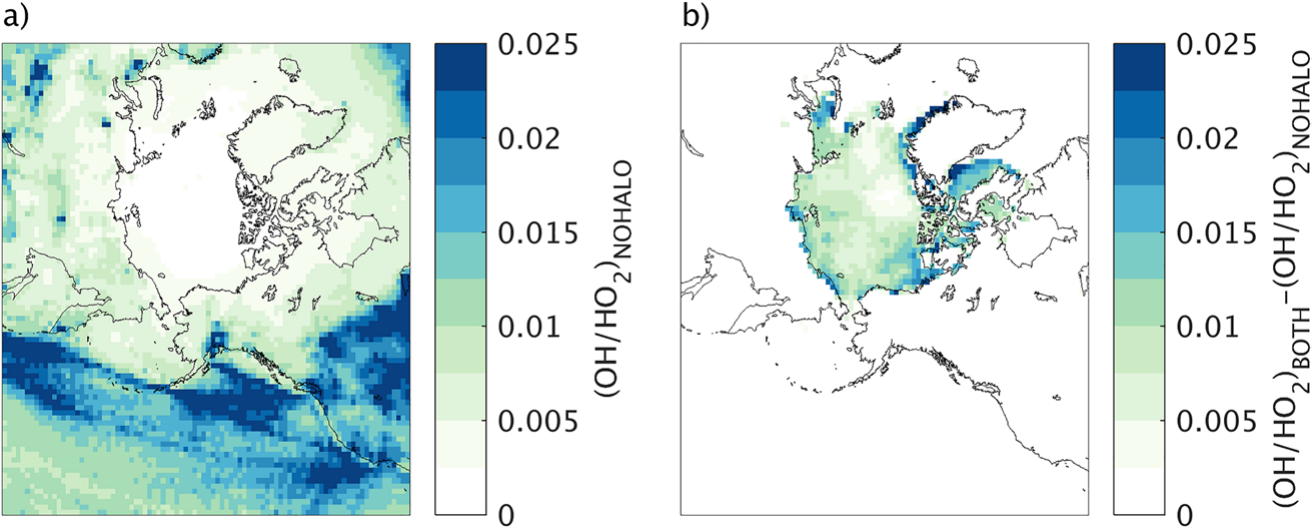
(a) OH/HO_2_ ratio in the simulation with no halogen chemistry and no updates implemented (NOHALO) run (b) increase in the OH/HO_2_ ratio upon including halogen chemistry, given as the difference between the BOTH and NOHALO runs: (OH/HO_2_)_BOTH_ ‐ (OH/HO_2_)_NOHALO_.

In order to show the impact of this chemistry for oxidation in the Arctic boundary layer, Figure 7a presents the OH/HO_2_ ratio for the NOHALO run, which is in the range of 0–0.03 for the Arctic and near 0 over most of the Arctic ocean. Figure 7b shows the difference in this ratio upon including halogen chemistry in the model. The OH/HO_2_ ratio increases by up to 0.03 over regions of the Arctic and Arctic Ocean upon including halogen chemistry. This is equal to the largest OH/HO_2_ ratio in the base run far from the Arctic Ocean. This indicates that the boundary layer over the Arctic Ocean may have oxidation conditions that are very different from most models at present, making it difficult to predict the lifetime of gas‐phase organics and aerosol precursors emitted from the Arctic Ocean during spring.

## 6 Spring 2012 in the Context of Meteorological Conditions and Past Studies

Our results indicate that surface snow was the main driver of ozone depletion events in the Arctic during Spring 2012. In agreement with previous work (Yang et al., 2019), we show that blowing snow has a strong impact on Arctic sea salt aerosol concentrations (supplementary Figure S1). However, in contradiction with previous work (Huang & Jaeglé, 2017; Huang et al., 2020; Yang et al., 2020), we find that blowing snow has little effect on Arctic ozone depletion, being responsible only for a few events and, regionally, at most for 10%–20% of the total depletion in a limited region along the Russian Coast. Here we explore some possible causes for these differences.

We think it is unlikely that our implementation underestimates blowing‐snow sourced Br_2_ emissions, since we found that sea salt aerosol emissions from blowing snow are likely overestimated in our implementation (supplementary Figure S1 and associated discussion). We used a high value of 0.38 for the depletion factor, meaning that 38% of all available bromine from these overestimated blowing snow sea salt emissions is emitted in our implementation. In addition, our study is to our knowledge the first to jointly assess model performance for surface meteorology, sea salt, ground based BrO, and surface ozone including central Arctic ozone, and none of these model/measurement comparisons indicate model deficiencies which could explain these differences.

Falk and Sinnhuber (2018) indicate that the surface snow mechanism leads observations by up to 2 days at Alert, Canada. In order to examine if blowing snow is better at reproducing the timing of the depletion events, rather than their magnitude, we also perform a time‐lagged correlation analysis (supplementary Figure S11). We do not find the same leading time lag than Falk and Sinnhuber (2018) at any coastal site nor at O‐buoy 6, where the max correlation is reached at or very near a 0 h time‐lag. We remind that the (0 h lag) correlation is always higher in SURFACE than in BLOWING, except for O‐buoy6 where the highest correlation is found for NOHALO. Since BLOWING has little effect on central Arctic ozone, it is closer to NOHALO and thus has a higher correlation than SURFACE at O‐buoy6, even though it is strongly biased against observations. At O‐buoy4, the correlation exhibits a maximum at −2 days for all simulations (SURFACE, NOHALO and BLOWING). Since this also occurs in NOHALO, this is most likely caused by a time lag in the meteorological reanalysis, and not by the implementation of the surface or blowing snow schemes. This is a known issue in the Arctic and especially in the central Arctic, where observations remain sparse and models, including reanalysis, have known problems reproducing meteorological systems.

We also examine if the lower role of blowing snow in ozone depletion found in our work could be due to meteorological differences in spring 2012 compared to other years. For example, in our simulations, wind speeds over Arctic sea ice, where salty blowing snow originates, are rarely above the critical threshold of 7 m/s (Supplementary Figure S4), and this threshold is exceeded even less often at the Arctic coastal sites (Supplementary Figure S5). We find (Supplementary Figure S9) that the blowing snow scheme emits sea salt aerosols as intended when this threshold is exceeded. Putting March‐April 2012 into the long‐term context, we find that wind speed was actually higher than normal over most of the Arctic (Supplementary Figure S8), which should in theory increase the influence of blowing snow in our simulations compared to earlier studies in other years. In addition, the Arctic surface was also mostly colder than average in March–April 2012 (Supplementary Figure S8), possibly indicating more stable conditions than usual, which could also intensify ODEs. Therefore, we conclude that it is unlikely that meteorological differences are to blame for the lower role of blowing snow in ozone depletion compared to earlier work.

In order to assess if 2012 was indeed a higher year in terms of bromine activity, we calculate the mean ozone concentration in March–April 2012 in Utqiagvik, and compare it to the long‐term March‐April mean for the 40 years 1973–2012. We also calculate the mean number of hours with depleted ozone (<10 ppbv) during the same March‐April period. Excluding the 10 years with insufficient data quality (more than 10% of missing data), we find that March–April 2012 was particularly active, with the second lowest mean ozone concentration in that record (12.2 ppbv, compared to a long‐term mean of 20.3 ppbv), and the largest number of hours of depleted ozone (817 h, compared to a long‐term average of 425 h). We think this high prevalence of ODEs in this season confirms that this period is particularly suited for investigating the origin of these events.

## 7 Conclusions

In this work, we have implemented descriptions of halogen chemistry, activation and recycling within the WRF‐Chem model. To our knowledge, this work is the first to implement both blowing snow and surface snow emissions of bromine into a single model, in order to compare their effects on springtime ozone depletion in the Arctic. We show that, in spring 2012, both bromine emission mechanisms can play a role in ozone depletion. Surface snow activation and recycling of bromine could be the key mechanism across most of the Arctic, while blowing snow could play an important role at specific sites and in initiating select events. We also show that the location of Br_2_ emissions are not necessarily correlated with either BrO or ozone depletion. Further, we show that including this chemistry significantly increases the OH/HO_2_ ratio at the surface regionally, especially over the Arctic Ocean.

Our results show, in agreement with previous studies, that blowing snow could be a strong source of sea salt aerosols over sea ice during spring. However, in contradiction with previous modeling work, we find that blowing snow has little effect on Arctic ozone depletion. We believe these differences can only be explained by completing a joint model study for the same time periods, using the same model input datasets (emissions, meteorology) and the same parameters, in order to compare the different components of the ozone and bromine budgets in these models. We think such a study will be extremely valuable to better understand the causes of Arctic ozone depletion, the sources of bromine, and to further improve models. The heterogeneous (including super‐cooled liquid and ice phase) chemistry of sea salt aerosols during Arctic spring as well as snow pack chemistry are both still uncertain despite existing studies (Edebeli et al., 2020; Huff & Abbatt, 2002; Hunt et al., 2004; Oum et al., 1998; Pratt et al., 2013). Further experiments under controlled lab conditions are needed to better understand bromine release from these surfaces in the future.

In the future, we also hope to investigate the relative roles of these processes in the Antarctic, where wind speeds are higher and blowing snow could be more important, and in other locations and years as new observations become available.

Our results provide a basis for future improvements in model predictions of surface ozone at the regional scale by improving the representation of Arctic halogen chemistry and determining the activation pathways of reactive bromine within WRF‐Chem. In the future, we aim to test how these mechanisms operate under past and future sea ice/snow cover conditions. Improved model predictions of polar halogen chemistry for ODEs and bromine activation events will allow us to better understand the oxidative processes for elemental mercury that lead to AMDEs and mercury deposition.

The functioning of atmospheric chemistry system in the lowest portion of the Arctic atmosphere may fundamentally change as the Arctic warms and ice and snow cover are reduced. Emissions from snow and ice will change as sea ice retreats, becomes thinner, more saline, and as snow on sea ice changes. Chemistry within the Arctic boundary layer determines the conditions that oceanic, ice, and snow emissions experience. Processes in the lowest portion of the atmosphere are also important because this is where species are most likely to be directly deposited back to the Arctic ocean, ice, and snow. It is only by developing predictive models that include halogen chemistry that we will be able to fully understand the impacts that future environmental changes (including sea ice change) and other anthropogenic influences will have within the Arctic region.

## Supporting information

Supporting Information S1

## Data Availability

The BROMEX measurements are currently being archived at https://asdc.larc.nasa.gov and currently available by contacting B. Simpson by email (wrsimpsonalaska.edu). The O‐buoy data is available from https://arcticdata.io/catalog/view/doi%3A10.18739%2FA2WD4W. The code used for this study is available on Zenodo as Marelle et al. (2021) https://doi.org/10.5281/zenodo.4607934.

## References

[jame21400-bib-0001] Abbatt, J. P. D., Thomas, J. L., Abrahamsson, K., Boxe, C., Granfors, A., Jones, A. E., et al. (2012). Halogen activation via interactions with environmental ice and snow in the polar lower troposphere and other regions. Atmospheric Chemistry and Physics, 12(14), 6237–6271. 10.5194/acp-12-6237-2012

[jame21400-bib-0002] Aguzzi, A., & Rossi, J. M. (1999). The kinetics of the heterogeneous reaction of BrONO_2_ with solid alkali halides at ambient temperature. A comparison with the interaction of ClONO_2_ on NaCl and KBr. Physical Chemistry Chemical Physics, 1, 4337–4346. 10.1039/A904611I

[jame21400-bib-0003] Ammann, M., Cox, R. A., Crowley, J. N., Jenkin, M. E., Mellouki, A., Rossi, M. J., et al. (2013). Evaluated kinetic and photochemical data for atmospheric chemistry: Volume VI – Heterogeneous reactions with liquid substrates. Atmospheric Chemistry and Physics, 13(16), 8045–8228. 10.5194/acp-13-8045-2013

[jame21400-bib-0004] Anderson, P. S., & Neff, W. D. (2008). Boundary layer physics over snow and ice. Atmospheric Chemistry and Physics, 8(13), 3563–3582. 10.5194/acp-8-3563-2008

[jame21400-bib-0005] Atkinson, R., Baulch, D. L., Cox, R. A., Crowley, J. N., Hampson, R. F., Hynes, R. G., et al. (2008). Evaluated kinetic and photochemical data for atmospheric chemistry: Volume IV – Gas phase reactions of organic halogen species. Atmospheric Chemistry and Physics, 8(15), 4141–4496. 10.5194/acp-8-4141-2008

[jame21400-bib-0006] Badia, A., Reeves, C. E., Baker, A. R., Saiz‐Lopez, A., Volkamer, R., Koenig, T. K., et al. (2019). Importance of reactive halogens in the tropical marine atmosphere: A regional modeling study using WRF‐Chem. Atmospheric Chemistry and Physics, 19(5), 3161–3189. 10.5194/acp-19-3161-2019

[jame21400-bib-0007] Barrie, L. A. (1986). Arctic air pollution: An overview of current knowledge. Atmospheric Environment, 20(4), 643–663. 10.1016/0004-6981(86)90180-0

[jame21400-bib-0008] Barrie, L. A., Bottenheim, J. W., Schnell, R. C., Crutzen, P. J., & Rasmussen, R. A. (1988). Ozone destruction and photochemical reactions at polar sunrise in the lower Arctic atmosphere. Nature, 334(6178), 138–141. 10.1038/334138a0

[jame21400-bib-0009] Berg, L. K., Shrivastava, M., Easter, R. C., Fast, J. D., Chapman, E. G., Liu, Y., & Ferrare, R. A. (2015). A new WRF‐Chem treatment for studying regional‐scale impacts of cloud processes on aerosol and trace gases in parameterized cumuli. Geoscientific Model Development, 8(2), 409–429. 10.5194/gmd-8-409-2015

[jame21400-bib-0010] Blechschmidt, A.‐M., Richter, A., Burrows, J. P., Kaleschke, L., Strong, K., Theys, N., et al. (2016). An exemplary case of a bromine explosion event linked to cyclone development in the Arctic. Atmospheric Chemistry and Physics, 16(3), 1773–1788. 10.5194/acp-16-1773-2016

[jame21400-bib-0011] Bougoudis, I., Blechschmidt, A.‐M., Richter, A., Seo, S., Burrows, J. P., Theys, N., & Rinke, A. (2020). Long‐term time‐series of Arctic Tropospheric BrO derived from UV‐VIS satellite remote sensing and its relation to first year sea ice. Atmospheric Chemistry and Physics Discussions, 2020, 1–38. 10.5194/acp-2020-116

[jame21400-bib-0012] Brioude, J., Arnold, D., Stohl, A., Cassiani, M., Morton, D., Seibert, P., et al. (2013). The Lagrangian particle dispersion model FLEXPART‐WRF version 3.1. Geoscientific Model Development, 6(6), 1889–1904. 10.5194/gmd-6-1889-2013

[jame21400-bib-0013] Burd, J. A., Peterson, P. K., Nghiem, S. V., Perovich, D. K., & Simpson, W. R. (2017). Snowmelt onset hinders bromine monoxide heterogeneous recycling in the Arctic. Journal of Geophysical Research: Atmosphere, 122(15), 8297–8309. 10.1002/2017jd026906

[jame21400-bib-0014] Burkholder, J., Sander, S., Abbatt, J., Barker, J., Huie, R., Kolb, C., et al. (2015). Chemical kinetics and photochemical data for use in atmospheric studies: Evaluation number 18 (tech. Rep.). Jet Propulsion Laboratory. Retrieved from http://jpldataeval.jpl.nasa.gov

[jame21400-bib-0015] Carter, W. P. L. (2000). *Documentation of the SAPRC‐99 chemical mechanism for VOC reactivity assessment. Final Report to California Air Resources Board Contract 92‐329 and Contract 95‐308*, (Tech. Rep.). Air Pollution Research Center and College of Engineering Center for Environmental Research and Technology, University of California Riverside.

[jame21400-bib-0016] Dee, D. P., Uppala, S. M., Simmons, A. J., Berrisford, P., Poli, P., Kobayashi, S., et al. (2011). The ERA‐Interim reanalysis: Configuration and performance of the data assimilation system. Quarterly Journal of the Royal Meteorological Society, 137(656), 553–597. 10.1002/qj.828

[jame21400-bib-0017] Deiber, G., George, C., Le Calvé, S., Schweitzer, F., & Mirabel, P. (2004). Uptake study of ClONO_2_ and BrONO_2_ by Halide containing droplets. Atmospheric Chemistry and Physics, 4(5), 1291–1299. 10.5194/acp-4-1291-2004

[jame21400-bib-0018] Dentener, F. J., & Crutzen, P. J. (1993). Reaction of N_2_O_5_ on tropospheric aerosols: Impact on the global distributions of NOx, O_3_, and OH. Journal of Geophysical Research, 98(D4), 7149–7163. 10.1029/92jd02979

[jame21400-bib-0019] Durre, I., Vose, R. S., & Wuertz, D. B. (2006). Overview of the integrated global radiosonde archive. Journal of Climate, 19(1), 53–68. 10.1175/JCLI3594.1

[jame21400-bib-0020] Edebeli, J., Trachsel, J. C., Avak, S. E., Ammann, M., Schneebeli, M., Eichler, A., & Bartels‐Rausch, T. (2020). Snow heterogeneous reactivity of bromide with ozone lost during snow metamorphism. Atmospheric Chemistry and Physics, 20(21), 13443–13454. 10.5194/acp-20-13443-2020

[jame21400-bib-0021] Emmons, L. K., Arnold, S. R., Monks, S. A., Huijnen, V., Tilmes, S., Law, K. S., et al. (2015). The POLARCAT Model Intercomparison Project (POLMIP): Overview and evaluation with observations. Atmospheric Chemistry and Physics, 15(12), 6721–6744. 10.5194/acp-15-6721-2015

[jame21400-bib-0022] Emmons, L. K., Walters, S., Hess, P. G., Lamarque, J.‐F., Pfister, G. G., Fillmore, D., et al. (2010). Description and evaluation of the Model for Ozone and Related chemical Tracers, version 4 (MOZART‐4). Geoscientific Model Development, 3(1), 43–67. 10.5194/gmd-3-43-2010

[jame21400-bib-0023] Falk, S., & Sinnhuber, B.‐M. (2018). Polar boundary layer bromine explosion and ozone depletion events in the chemistry‐climate model EMAC v2.52: Implementation and evaluation of AirSnow algorithm. Geoscientific Model Development, 11(3), 1115–1131. 10.5194/gmd-11-1115-2018

[jame21400-bib-0024] Fast, J. D., Gustafson, W. I., Easter, R. C., Zaveri, R. A., Barnard, J. C., Chapman, E. G., et al. (2006). Evolution of ozone, particulates, and aerosol direct radiative forcing in the vicinity of Houston using a fully coupled meteorology‐chemistry‐aerosol model. Journal of Geophysical Research, 111(D21). 10.1029/2005JD006721

[jame21400-bib-0025] Fernandez, R. P., Carmona‐Balea, A., Cuevas, C. A., Barrera, J. A., Kinnison, D. E., Lamarque, J.‐F., et al. (2019). Modeling the sources and chemistry of polar tropospheric Halogens (Cl, Br, and I) using the CAM‐Chem global chemistry‐climate model. Journal of Advances in Modeling Earth Systems, 11(7), 2259–2289. 10.1029/2019ms001655

[jame21400-bib-0027] Frey, M., Norris, S., Brooks, I., Anderson, P., Nishimura, K., Yang, X., et al. (2020). First direct observation of sea salt aerosol production from blowing snow above sea ice. Atmospheric Chemistry and Physics, 20, 2549–2578. 10.5194/acp-20-2549-2020

[jame21400-bib-0026] Frey, M. M., & Nomura, D. (2019). Salinity profiles of snow on sea ice and sea ice in the Arctic Ocean during winter 2015. & UK Polar Data Centre, Natural Environment Research Council. 10.5285/6ed9f4ea-6b89-4059-84e3-5c4118b68db9

[jame21400-bib-0028] Fuchs, N. A., & Sutugin, A. G. (1971). High‐dispersed aerosols. In G. M. Hidy, & J. R. Brock (Eds.), Topics in current aerosol research (part 2). Pergamon. 10.1016/B978-0-08-016674-2.50006-6

[jame21400-bib-0029] Gong, S. L., Barrie, L. A., & Blanchet, J.‐P. (1997). Modeling sea‐salt aerosols in the atmosphere: 1. model development. Journal of Geophysical Research, 102(D3), 3805–3818. 10.1029/96jd02953

[jame21400-bib-0030] Gratz, L. E., Ambrose, J. L., Jaffe, D. A., Shah, V., Jaeglé, L., Stutz, J., et al. (2015). Oxidation of mercury by bromine in the subtropical Pacific free troposphere. Geophysical Research Letters, 42(23), 10494–10502. 10.1002/2015gl066645

[jame21400-bib-0031] Grell, G. A., Peckham, S. E., Schmitz, R., McKeen, S. A., Frost, G., Skamarock, W. C., & Eder, B. (2005). Fully coupled “online” chemistry within the WRF model. Atmospheric Environment, 39(37), 6957–6975. 10.1016/j.atmosenv.2005.04.027

[jame21400-bib-0032] Halfacre, J. W., Knepp, T. N., Shepson, P. B., Thompson, C. R., Pratt, K. A., Li, B., et al. (2014). Temporal and spatial characteristics of ozone depletion events from measurements in the arctic. Atmospheric Chemistry and Physics, 14(10), 4875–4894. 10.5194/acp-14-4875-2014

[jame21400-bib-0033] Herrmann, M., Sihler, H., Frieß, U., Wagner, T., Platt, U., & Gutheil, E. (2021). Time‐dependent 3d simulations of tropospheric ozone depletion events in the arctic spring using the weather research and forecasting model coupled with chemistry (wrf‐chem). Atmospheric Chemistry and Physics, 21(10), 7611–7638. 10.5194/acp-21-7611-2021

[jame21400-bib-0034] Hong, S.‐Y., Noh, Y., & Dudhia, J. (2006). A new vertical diffusion package with an explicit treatment of entrainment processes. Monthly Weather Review, 134(9), 2318–2341. 10.1175/MWR3199.1

[jame21400-bib-0035] Huang, J., & Jaeglé, L. (2017). Wintertime enhancements of sea salt aerosol in Polar regions consistent with a sea ice source from blowing snow. Atmospheric Chemistry and Physics, 17(5), 3699–3712. 10.5194/acp-17-3699-2017

[jame21400-bib-0036] Huang, J., Jaeglé, L., Chen, Q., Alexander, B., Sherwen, T., Evans, M. J., et al. (2020). Evaluating the impact of blowing‐snow sea salt aerosol on springtime BrO and O_3_ in the Arctic. Atmospheric Chemistry and Physics, 20(12), 7335–7358. 10.5194/acp-20-7335-2020

[jame21400-bib-0037] Huang, J., Jaeglé, L., & Shah, V. (2018). Using CALIOP to constrain blowing snow emissions of sea salt aerosols over Arctic and Antarctic sea ice. Atmospheric Chemistry and Physics, 18(22), 16253–16269. 10.5194/acp-18-16253-2018

[jame21400-bib-0038] Huff, A. K., & Abbatt, J. P. D. (2002). Kinetics and product yields in the heterogeneous reactions of hobr with ice surfaces containing nabr and nacl. The Journal of Physical Chemistry A, 106(21), 5279–5287. 10.1021/jp014296m

[jame21400-bib-0039] Hunt, S. W., Roeselová, M., Wang, W., Wingen, L. M., Knipping, E. M., Tobias, D. J., et al. (2004). Formation of molecular bromine from the reaction of ozone with deliquesced nabr aerosol: Evidence for interface chemistry. The Journal of Physical Chemistry A, 108(52), 11559–11572. 10.1021/jp0467346

[jame21400-bib-0040] Iacono, M. J., Delamere, J. S., Mlawer, E. J., Shephard, M. W., Clough, S. A., & Collins, W. D. (2008). Radiative forcing by long‐lived greenhouse gases: Calculations with the AER radiative transfer models. Journal of Geophysical Research, 113(D13). 10.1029/2008jd009944

[jame21400-bib-0041] International Union of Pure and Applied Chemistry . (2009). *IUPAC Task Group on Atmospheric Chemical Kinetic Data Evaluation*. Retrieved from http://iupac.pole-ether.fr

[jame21400-bib-0042] Janjić, Z. I. (1994). The step‐mountain eta coordinate model: Further developments of the convection, viscous sublayer, and turbulence closure schemes. Monthly Weather Review, 122(5), 927–945. 10.1175/1520-0493(1994)122<0927:TSMECM>2.0.CO;2

[jame21400-bib-0043] Jones, A. E., Anderson, P. S., Begoin, M., Brough, N., Hutterli, M. A., Marshall, G. J., et al. (2009). BrO, blizzards, and drivers of polar tropospheric ozone depletion events. Atmospheric Chemistry and Physics, 9(14), 4639–4652. 10.5194/acp-9-4639-2009

[jame21400-bib-0044] Knepp, T. N., Bottenheim, J., Carlsen, M., Carlson, D., Donohoue, D., Friederich, G., et al. (2010). Development of an autonomous sea ice tethered buoy for the study of ocean‐atmosphere‐sea ice‐snow pack interactions: The o‐buoy. Atmospheric Measurement Techniques, 3(1), 249–261. 10.5194/amt-3-249-2010

[jame21400-bib-0045] Knipping, E. M., Lakin, M. J., Foster, K. L., Jungwirth, P., Tobias, D. J., Gerber, R. B., et al. (2000). Experiments and simulations of ion‐enhanced interfacial chemistry on aqueous NaCl aerosols. Science, 288(5464), 301–306. 10.1126/science.288.5464.301 10764637

[jame21400-bib-0046] Laskin, A., Wang, H., Robertson, W. H., Cowin, J. P., Ezell, M. J., & Finlayson‐Pitts, B. J. (2006). A new approach to determining gas‐particle reaction probabilities and application to the heterogeneous reaction of deliquesced sodium chloride particles with gas‐phase hydroxyl radicals. The Journal of Physical Chemistry A, 110(36), 10619–10627. 10.1021/jp063263+ 16956244

[jame21400-bib-0047] Liao, J., Huey, L. G., Tanner, D. J., Brough, N., Brooks, S., Dibb, J. E., et al. (2011). Observations of hydroxyl and peroxy radicals and the impact of BrO at Summit, Greenland in 2007 and 2008. Atmospheric Chemistry and Physics, 11(16), 8577–8591. 10.5194/acp-11-8577-2011

[jame21400-bib-0048] Marelle, L., Raut, J.‐C., Law, K. S., Berg, L. K., Fast, J. D., Easter, R. C., et al. (2017). Improvements to the WRF‐Chem 3.5.1 model for quasi‐hemispheric simulations of aerosols and ozone in the Arctic. Geoscientific Model Development, 10(10), 3661–3677. 10.5194/gmd-10-3661-2017

[jame21400-bib-0049] Marelle, L., Thomas, J. L., Ahmed, S., Tuite, K., Stutz, J., Dommergue, A., et al. (2021). *WRF‐Chem 4.1.1 version including polar bromine chemistry and emissions*. Zenodo. 10.5281/zenodo.4607934

[jame21400-bib-0050] Massom, R. A., Eicken, H., Hass, C., Jeffries, M. O., Drinkwater, M. R., Sturm, M., et al. (2001). Snow on Antarctic sea ice. Reviews of Geophysics, 39(3), 413–445. 10.1029/2000rg000085

[jame21400-bib-0051] Monks, S. A., Arnold, S. R., Emmons, L. K., Law, K. S., Turquety, S., Duncan, B. N., et al. (2015). Multi‐model study of chemical and physical controls on transport of anthropogenic and biomass burning pollution to the Arctic. Atmospheric Chemistry and Physics, 15(6), 3575–3603. 10.5194/acp-15-3575-2015

[jame21400-bib-0052] Morrison, H., Thompson, G., & Tatarskii, V. (2009). Impact of cloud microphysics on the development of trailing stratiform precipitation in a simulated squall line: Comparison of one‐ and two‐moment schemes. Monthly Weather Review, 137(3), 991–1007. 10.1175/2008MWR2556.1

[jame21400-bib-0053] Nakanishi, M., & Niino, H. (2009). Development of an improved turbulence closure model for the atmospheric doundary layer. Journal of the Meteorological Society of Japan, 87(5), 895–912. 10.2151/jmsj.87.895

[jame21400-bib-0054] National Centers for Environmental Prediction . (2000). *NCEP FNL operational model global tropospheric analyses*. Research Data Archive at the National Center for Atmospheric Research, Computational and Information Systems Laboratory. 10.5065/D6M043C6

[jame21400-bib-0055] Niu, G.‐Y., Yang, Z.‐L., Mitchell, K. E., Chen, F., Ek, M. B., Barlage, M., et al. (2011). The community Noah land surface model with multiparameterization options (Noah‐MP): 1. Model description and evaluation with local‐scale measurements. Journal of Geophysical Research, 116(D12), D12109. 10.1029/2010jd015139

[jame21400-bib-0056] Oleson, K. W., Lawrence, D. M., Bonan, G. B., Flanner, M. G., Kluzek, E., Lawrence, P. J., et al. (2010). *Technical description of version 4.0 of the community land model (CLM)*.

[jame21400-bib-0057] Oltmans, S. J. (1981). Surface ozone measurements in clean air. Journal of Geophysical Research, 86(C2), 1174–1180. 10.1029/JC086iC02p01174

[jame21400-bib-0058] Oum, K. W., Lakin, M. J., & Finlayson‐Pitts, B. J. (1998). Bromine activation in the troposphere by the dark reaction of O3 with seawater ice. Geophysical Research Letters, 25(21), 3923–3926. 10.1029/1998gl900078

[jame21400-bib-0059] Peterson, P. K., Hartwig, M., May, N. W., Schwartz, E., Rigor, I., Ermold, W., et al. (2019). Snowpack measurements suggest role for multi‐year sea ice regions in Arctic atmospheric bromine and chlorine chemistry. Elementa Science of the Anthropocene, 7(14). 10.1525/elementa.352 PMC6750228 31534978

[jame21400-bib-0060] Peterson, P. K., Pöhler, D., Sihler, H., Zielcke, J., General, S., Frie, U., et al. (2017). Observations of bromine monoxide transport in the Arctic sustained on aerosol particles. Atmospheric Chemistry and Physics, 17(12), 7567–7579. 10.5194/acp-17-7567-2017

[jame21400-bib-0061] Peterson, P. K., Simpson, W. R., Pratt, K. A., Shepson, P. B., Frieß, U., Zielcke, J., et al. (2015). Dependence of the vertical distribution of bromine monoxide in the lower troposphere on meteorological factors such as wind speed and stability. Atmospheric Chemistry and Physics, 15(4), 2119–2137. 10.5194/acp-15-2119-2015

[jame21400-bib-0062] Piot, M., & Glasow, R. V. (2009). Modeling the multiphase near‐surface chemistry related to ozone depletions in polar spring. Journal of Atmospheric Chemistry, 64(2), 77–105. 10.1007/s10874-010-9170-1

[jame21400-bib-0063] Platt, U., & Hönninger, G. (2003). The role of halogen species in the troposphere. Chemosphere, 52(2), 325–338. 10.1016/S0045-6535(03)00216-9 12738256

[jame21400-bib-0064] Pratt, K. A., Custard, K. D., Shepson, P. B., Douglas, T. A., Pöhler, D., General, S., et al. (2013). Photochemical production of molecular bromine in Arctic surface snowpacks. Nature Geoscience, 6(5), 351–356. 10.1038/ngeo1779

[jame21400-bib-0065] Pratte, P., & Rossi, M. J. (2006). The heterogeneous kinetics of HOBr and HOCl on acidified sea salt and model aerosol at 40–90% relative humidity and ambient temperature. Physical Chemistry Chemical Physics, 8, 3988–4001. 10.1039/B604321F 17028689

[jame21400-bib-0066] Provost, C., Sennéchael, N., Miguet, J., Itkin, P., Rösel, A., Koenig, Z., et al. (2017). Observations of flooding and snow‐ice formation in a thinner arctic sea‐ice regime during the n‐ice2015 campaign: Influence of basal ice melt and storms. Journal of Geophysical Research: Oceans, 122(9), 7115–7134. 10.1002/2016jc012011

[jame21400-bib-0067] Rhodes, R. H., Yang, X., Wolff, E. W., McConnell, J. R., & Frey, M. M. (2017). Sea ice as a source of sea salt aerosol to Greenland ice cores: A model‐based study. Atmospheric Chemistry and Physics, 17(15), 9417–9433. 10.5194/acp-17-9417-2017

[jame21400-bib-0068] Sander, R. (2015). Compilation of Henry's law constants (version 4.0) for water as solvent. Atmospheric Chemistry and Physics, 15(8), 4399–4981. 10.5194/acp-15-4399-2015

[jame21400-bib-0069] Sander, R., Keene, W. C., Pszenny, A. A. P., Arimoto, R., Ayers, G. P., Baboukas, E., et al. (2003). Inorganic bromine in the marine boundary layer: A critical review. Atmospheric Chemistry and Physics, 3(5), 1301–1336. 10.5194/acp-3-1301-2003

[jame21400-bib-0070] Seinfeld, J. H., & Pandis, S. N. (1998). Atmospheric Chemistry and Physics: From air pollution to climate change. John Wiley & Sons.

[jame21400-bib-0071] Seisel, S., Flückiger, B., & Rossi, M. J. (1998). The heterogeneous reaction of N_2_O_5_ with HBr on Ice comparison with N_2_O_5_+HCl. Berichte der Bunsen‐Gesellschaft für Physikalische Chemie, 102(6), 811–820. 10.1002/bbpc.19981020604

[jame21400-bib-0072] Simpson, W. R., Brown, S. S., Saiz‐Lopez, A., Thornton, J. A., & von Glasow, R. (2015). Tropospheric halogen chemistry: Sources, cycling, and impacts. Chemical Reviews, 115(10), 4035–4062. 10.1021/cr5006638 25763598 PMC4469175

[jame21400-bib-0073] Simpson, W. R., Carlson, D., Hönninger, G., Douglas, T. A., Sturm, M., Perovich, D., & Platt, U. (2007). First‐year sea‐ice contact predicts bromine monoxide (BrO) levels at Barrow, Alaska better than potential frost flower contact. Atmospheric Chemistry and Physics, 7(3), 621–627. 10.5194/acp-7-621-2007

[jame21400-bib-0074] Simpson, W. R., Perovich, D., Matrai, P., Shepson, P., & Chavez, F. (2009). *The collaborative O‐buoy project: Deployment of a network of Arctic ocean chemical sensors for the IPY and beyond*. Arctic data center. 10.18739/A2WD4W

[jame21400-bib-0075] Simpson, W. R., Peterson, P., Frieß, U., Sihler, H., Lampel, J., Platt, U., et al. (2017). Horizontal and vertical structure of reactive bromine events probed by bromine monoxide MAX‐DOAS. Atmospheric Chemistry and Physics, 17(15), 9291–9309. 10.5194/acp-17-9291-2017

[jame21400-bib-0076] Simpson, W. R., von Glasow, R., Riedel, K., Anderson, P., Ariya, P., Bottenheim, J., et al. (2007). Halogens and their role in polar boundary‐layer ozone depletion. Atmospheric Chemistry and Physics, 7(16), 4375–4418. 10.5194/acp-7-4375-2007

[jame21400-bib-0077] Spolaor, A., Vallelonga, P., Plane, J. M. C., Kehrwald, N., Gabrieli, J., Varin, C., et al. (2013). Halogen species record Antarctic sea ice extent over glacial–interglacial periods. Atmospheric Chemistry and Physics, 13(13), 6623–6635. 10.5194/acp-13-6623-2013

[jame21400-bib-0078] Spolaor, A., Vallelonga, P., Turetta, C., Maffezzoli, N., Cozzi, G., Gabrieli, J., et al. (2016). Canadian Arctic sea ice reconstructed from bromine in the Greenland NEEM ice core. Scientific Reports, 6(1), 33925. 10.1038/srep33925 27650478 PMC5030631

[jame21400-bib-0079] Sterk, H. A. M., Steeneveld, G. J., Vihma, T., Anderson, P. S., Bosveld, F. C., & Holtslag, A. A. M. (2015). Clear‐sky stable boundary layers with low winds over snow‐covered surfaces. part 1: Wrf model evaluation. Quarterly Journal of the Royal Meteorological Society, 141(691), 2165–2184. 10.1002/qj.2513

[jame21400-bib-0080] Stohl, A., Forster, C., Frank, A., Seibert, P., & Wotawa, G. (2005). Technical note: The Lagrangian particle dispersion model FLEXPART version 6.2. Atmospheric Chemistry and Physics, 5(9), 2461–2474. 10.5194/acp-5-2461-2005

[jame21400-bib-0081] Stutz, J., Thomas, J. L., Hurlock, S. C., Schneider, M., von Glasow, R., Piot, M., et al. (2011). Longpath DOAS observations of surface BrO at Summit, Greenland. Atmospheric Chemistry and Physics, 11(18), 9899–9910. 10.5194/acp-11-9899-2011

[jame21400-bib-0082] Swanson, W. F., Graham, K. A., Halfacre, J. W., Holmes, C. D., Shepson, P. B., & Simpson, W. R. (2020). Arctic reactive bromine events occur in two distinct sets of environmental conditions: A statistical analysis of 6 years of observations. Journal of Geophysical Research: Atmosphere, 125(10), e2019JD032139. 10.1029/2019jd032139

[jame21400-bib-0083] Tewari, M., Chen, F., Wang, W., Dudhia, J., LeMone, M. A., Gayno, G., et al. (2004). Implementation and verification of the unified Noah land surface model in the WRF model. In 20th conference on weather analysis and forecasting/16th conference on numerical weather prediction. (pp. 11–15).

[jame21400-bib-0084] Thomas, J. L., Dibb, J. E., Huey, L. G., Liao, J., Tanner, D., Lefer, B., et al. (2012). Modeling chemistry in and above snow at Summit, Greenland – Part 2: Impact of snowpack chemistry on the oxidation capacity of the boundary layer. Atmospheric Chemistry and Physics, 12(14), 6537–6554. 10.5194/acp-12-6537-2012

[jame21400-bib-0085] Thomas, J. L., Dibb, J. E., Stutz, J., von Glasow, R., Brooks, S., Huey, L. G., & Lefer, B. (2012). Overview of the 2007 and 2008 campaigns conducted as part of the Greenland Summit Halogen‐HOx Experiment (GSHOX). Atmospheric Chemistry and Physics, 12(22), 10833–10839. 10.5194/acp-12-10833-2012

[jame21400-bib-0086] Thomas, J. L., Stutz, J., Lefer, B., Huey, L. G., Toyota, K., Dibb, J. E., & von Glasow, R. (2011). Modeling chemistry in and above snow at Summit, Greenland – Part 1: Model description and results. Atmospheric Chemistry and Physics, 11(10), 4899–4914. 10.5194/acp-11-4899-2011

[jame21400-bib-0088] Toyota, K., McConnell, J. C., Staebler, R. M., & Dastoor, A. P. (2014). Air–snowpack exchange of bromine, ozone and mercury in the springtime Arctic simulated by the 1‐D model PHANTAS ‐ Part 1: In‐snow bromine activation and its impact on ozone. Atmospheric Chemistry and Physics, 14(8), 4101–4133. 10.5194/acp-14-4101-2014

[jame21400-bib-0087] Toyota, K., McConnell, J., Lupu, A., Neary, L., Mclinden, C., Richter, A., et al. (2011). Analysis of reactive bromine production and ozone depletion in the Arctic boundary layer using 3‐D simulations with GEM‐AQ: Inference from synoptic‐scale patterns. Atmospheric Chemistry and Physics, 11(8), 3949–3979. 10.5194/acp-11-3949-2011

[jame21400-bib-0089] von Glasow, R., Sander, R., Bott, A., & Crutzen, P. J. (2002a). Modeling halogen chemistry in the marine boundary layer 1. Cloud‐free MBL. Journal of Geophysical Research, 107(D17), 4341. 10.1029/2001jd000942

[jame21400-bib-0090] von Glasow, R., Sander, R., Bott, A., & Crutzen, P. J. (2002b). Modeling halogen chemistry in the marine boundary layer 2. Interactions with sulfur and the cloud‐covered MBL. Journal of Geophysical Research, 107(D17). 10.1029/2001jd000943

[jame21400-bib-0091] Wang, C., Graham, R. M., Wang, K., Gerland, S., & Granskog, M. A. (2019). Comparison of era5 and era‐interim near‐surface air temperature, snowfall and precipitation over arctic sea ice: Effects on sea ice thermodynamics and evolution. The Cryosphere, 13(6), 1661–1679. 10.5194/tc-13-1661-2019

[jame21400-bib-0092] Wang, S., McNamara, S. M., Moore, C. W., Obrist, D., Steffen, A., Shepson, P. B., et al. (2019). Direct detection of atmospheric atomic bromine leading to mercury and ozone depletion. Proceedings of the National Academy of Sciences, 116(29), 14479–14484. 10.1073/pnas.1900613116 PMC6642345 31253702

[jame21400-bib-0093] Wesely, M. (1989). Parameterization of surface resistances to gaseous dry deposition in regional‐scale numerical models. Atmospheric Environment, 23(6), 1293–1304. 10.1016/0004-6981(89)90153-4

[jame21400-bib-0094] Wild, O., Zhu, X., & Prather, M. J. (2000). Fast‐J: Accurate simulation of in‐ and below‐cloud photolysis in tropospheric chemical models. Journal of Atmospheric Chemistry, 37(3), 245–282. 10.1023/A:1006415919030

[jame21400-bib-0095] Yang, X., Blechschmidt, A.‐M., Bognar, K., McClure‐Begley, A., Morris, S., Petropavlovskikh, I., et al. (2020). Pan‐Arctic surface ozone: Modeling vs measurements. Atmospheric Chemistry and Physics Discussions, 2020, 1–33. 10.5194/acp-2019-984

[jame21400-bib-0096] Yang, X., Frey, M. M., Rhodes, R. H., Norris, S. J., Brooks, I. M., Anderson, P. S., et al. (2019). Sea salt aerosol production via sublimating wind‐blown saline snow particles over sea ice: Parameterizations and relevant microphysical mechanisms. Atmospheric Chemistry and Physics, 19(13), 8407–8424. 10.5194/acp-19-8407-2019

[jame21400-bib-0097] Yang, X., Pyle, J. A., & Cox, R. A. (2008). Sea salt aerosol production and bromine release: Role of snow on sea ice. Geophysical Research Letters, 35(16). 10.1029/2008GL034536

[jame21400-bib-0098] Yang, X., Pyle, J. A., Cox, R. A., Theys, N., & Van Roozendael, M. (2010). Snow‐sourced bromine and its implications for polar tropospheric ozone. Atmospheric Chemistry and Physics, 10(16), 7763–7773. 10.5194/acp-10-7763-2010

[jame21400-bib-0099] Zaveri, R. A., Easter, R. C., Fast, J. D., & Peters, L. K. (2008). Model for simulating aerosol interactions and chemistry (MOSAIC). Journal of Geophysical Research, 113(D13), D13204. 10.1029/2007JD008782

