# Supplementary material for: Implementation and Impacts of Surface and Blowing Snow Sources of Arctic Bromine Activation Within WRF‐Chem 4.1.1
Source: J Adv Model Earth Syst. 2021 Jul 30;13(8):e2020MS002391. doi: 10.1029/2020MS002391 (PMC8365729; doi:10.1029/2020MS002391)
Supplement: Supplementary file 1 — Supporting Information S1 [file JAME-13-e2020MS002391-s001.docx]

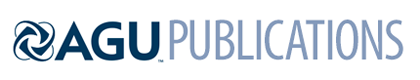


*Journal of Advances in Modeling Earth Systems (JAMES)*

Supporting Information for

**Implementation and impacts of surface and blowing snow sources of Arctic bromine activation within WRF-Chem 4.1.1**

Louis Marelle^1,2^, Jennie L. Thomas^1^, Shaddy Ahmed^1^, Katie Tuite^3^, Jochen Stutz^3^, Aurélien Dommergue^1^, William R. Simpson^4^, Markus M. Frey^5^, Foteini Baladima^1^

^1^Université de Grenoble Alpes, CNRS, IRD, IGE, Grenoble, France

^2^LATMOS/IPSL, Sorbonne Université, UVSQ, CNRS, Paris, France

^3^Department of Atmospheric and Oceanic Sciences, University of California, Los Angeles, CA, USA

^4^Geophysical Institute and Department of Chemistry and Biochemistry, University of Alaska Fairbanks, Fairbanks, AK, USA

^5^British Antarctic Survey, Natural Environment Research Council, Cambridge, UK

**Contents of this file**

Figures S1 to S11 and Table S1

**Introduction**

This supporting information includes 11 Figures and 1 Table. The methods used to generate each supplementary element is explained in their respective captions.

Specifically, Figure S1 compares the concentrations of sea salt aerosol sodium (Na+) concentrations observed in surface air at Nord, Greenland; Alert, Canada; Zeppelin, Svalbard to WRF-Chem simulation results. The main purpose of this Figure is to compare the results from the BLOWING simulation presented in the manuscript to the results of simulation BLOWING_Y2008, which uses the original blowing snow scheme parameters from Yang et al. (2008), including a higher snow salinity (snow salinity distribution of mean 8.3 psu) and N=1 aerosol fractioning. The Figure also includes 2 other sensitivity blowing snow simulations using N=5 and a low and high estimate for Arctic snow salinity: 0.01 psu (BLOWING_0.01PSU) and 1.7 psu (BLOWING_1.7PSU). The sea salt sodium data was obtained from EBAS (http://ebas.nilu.no/)

Figures S2 and S3 follow the methods used to produce the corresponding Figures 3 and 4 in the main text, following the BOTH simulation description, but with variable fractional sea ice treatment within the halogen chemistry activation mechanisms. Specifically we use different sea ice fraction thresholds for which blowing snow emissions of Br_2_ and sea salt, surface snow emissions of Br_2_, and surface recycling processes are triggered. In each simulation, these are active for sea ice above these factional sea ice thresholds. Based on these simulations, we use the 75% threshold for all simulations presented in the main text of the paper. Compared to the implementation of BOTH presented in the manuscript, these simulations also include blowing snow emissions with the original parameters from Yang et al. (2008), including a higher salinity (snow salinity distribution of mean 8.3 psu) and N=1 aerosol fractioning.

Figure S4 shows the frequency that the critical threshold for lofting blowing snow was exceeded over the entire model domain. This was calculated as a percentage of times when 10-meter wind speeds were above 7 m s^-1^ in each grid cell between 08/03/2012 and 01/05/2012. The sea ice extent, obtained from the Multisensor Analyzed Sea Ice Extent - Northern Hemisphere (MASIE-NH), is also plotted (in green, for April 15 2012) to show the frequency the critical threshold was exceeded over sea ice.

Reference for MAISE-NH dataset:

*U.S. National Ice Center and National Snow and Ice Data Center. Compiled by F. Fetterer, M. Savoie, S. Helfrich, and P. Clemente-Colón. 2010, updated daily. Multisensor Analyzed Sea Ice Extent - Northern Hemisphere (MASIE-NH), Version 1. 4 km x 4 km. Boulder, Colorado USA. NSIDC: National Snow and Ice Data Center. doi:*[*https://doi.org/10.7265/N5GT5K3K*](https://www.google.com/url?q=https://doi.org/10.7265/N5GT5K3K&sa=D&source=editors&ust=1615844341157000&usg=AOvVaw2YJctCjgVVv4omwYMAwHZU)*. Date Accessed: 05 February 2021.*

Figure S5 shows a timeseries plot for the model calculated 10-meter wind speeds at the 4 coastal Arctic stations used in this work.

Figures S6 and S7 follow the methods used to produce the corresponding Figure 6, but show the absolute values (Figure S6) and the relative difference e.g. SURFACE-NOHALO/BOTH-NOHALO (Figure S7) instead of the e.g. SURFACE-NOHALO absolute difference shown in Figure 6.

Figure S8 presents the March-April 2012 mean surface wind speed and surface temperature from the NCEP reanalysis, compared to the long-term March-April 2000-2019 mean from the same dataset.

Reference for the NCEP reanalysis dataset:

*National Centers for Environmental Prediction/National Weather Service/NOAA/U.S. Department of Commerce. 2000. NCEP/DOE Reanalysis 2 (R2). Research Data Archive at the National Center for Atmospheric Research, Computational and Information Systems Laboratory. https://doi.org/10.5065/KVQZ-YJ93. Accessed 1 March 2021.*

Figure S9 compares for 1 March 2012 the frequency of WRF-Chem 10-m windspeed over 7m/s (the approximate threshold for lifting snow) to the daily averaged WRF-Chem sea salt aerosol concentration from blowing snow only during that day(BLOWING-NOHALO). The Figure shows blowing snow emits sea salt in the areas of the ice pack where the 7m/s is exceeded.

Figure S10 is the same than manuscript Figure 4, but also includes modeled ozone evaluation from to the sensitivity blowing snow simulations BLOWING_0.01PSU and BLOWING_1.7PSU.

Figure S11 shows a time-lagged correlation analysis between observed and modeled ozone (NOHALO, SURFACE, BLOWING, BOTH) at the 6 Arctic surface sites (using the data presented on manuscript Figure 4).


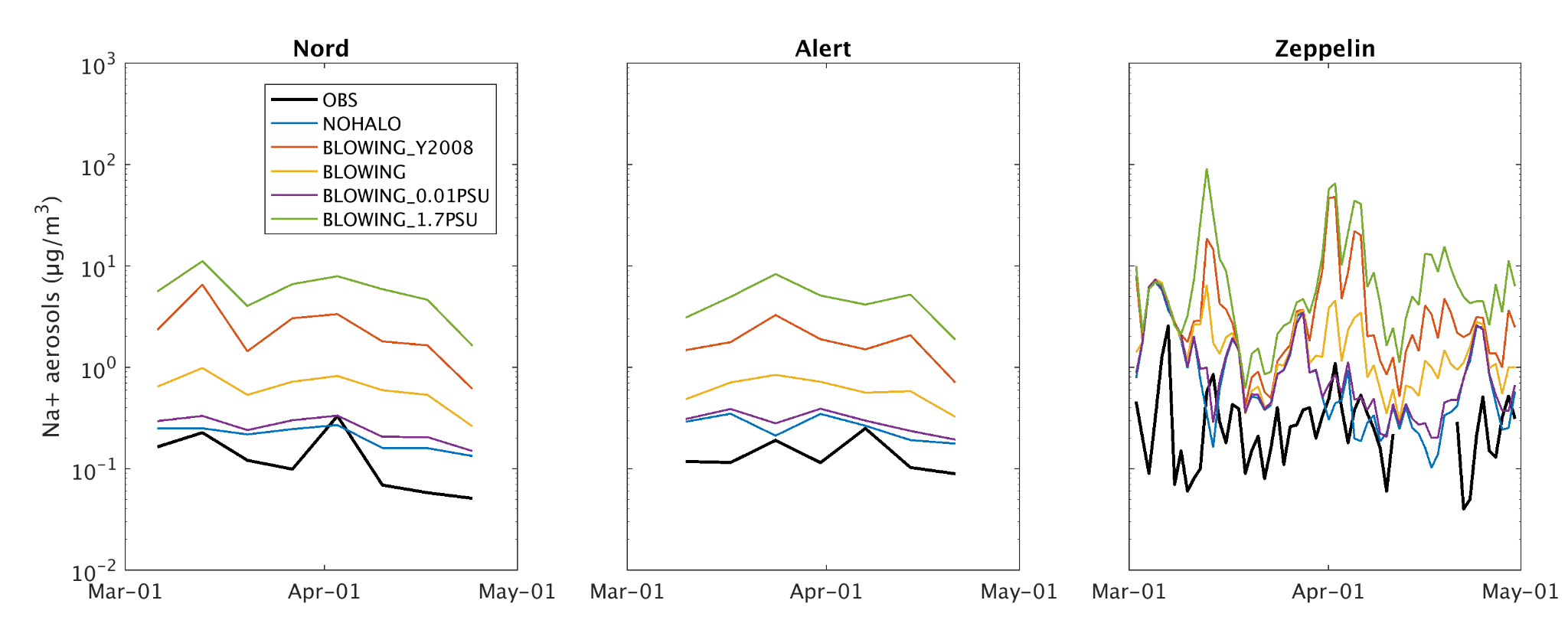


**Figure S1.** Evaluation of model predicted sea salt (Na mass) at three Arctic sites for the no halogen run (NOHALO, with only open ocean sea salt emissions), and 4 different blowing snow schemes using different values for the parameters: the original Yang et al. (2008) scheme (BLOWING_Y2008, using N=1 and salinity from Massom et al., 2001), the updated parameters in the manuscript (BLOWING, with 0.1 psu salinity and N=5), and additional sensitivity simulations with N=5 and snow salinities of 0.01 psu and 1.7 psu (BLOWING_0.01PSU and BLOWING_1.7PSU). The model consistently over predicts sea salt aerosols, while the updated BLOWING and BLOWING_0.01PSU runs are in better agreement than the original version.


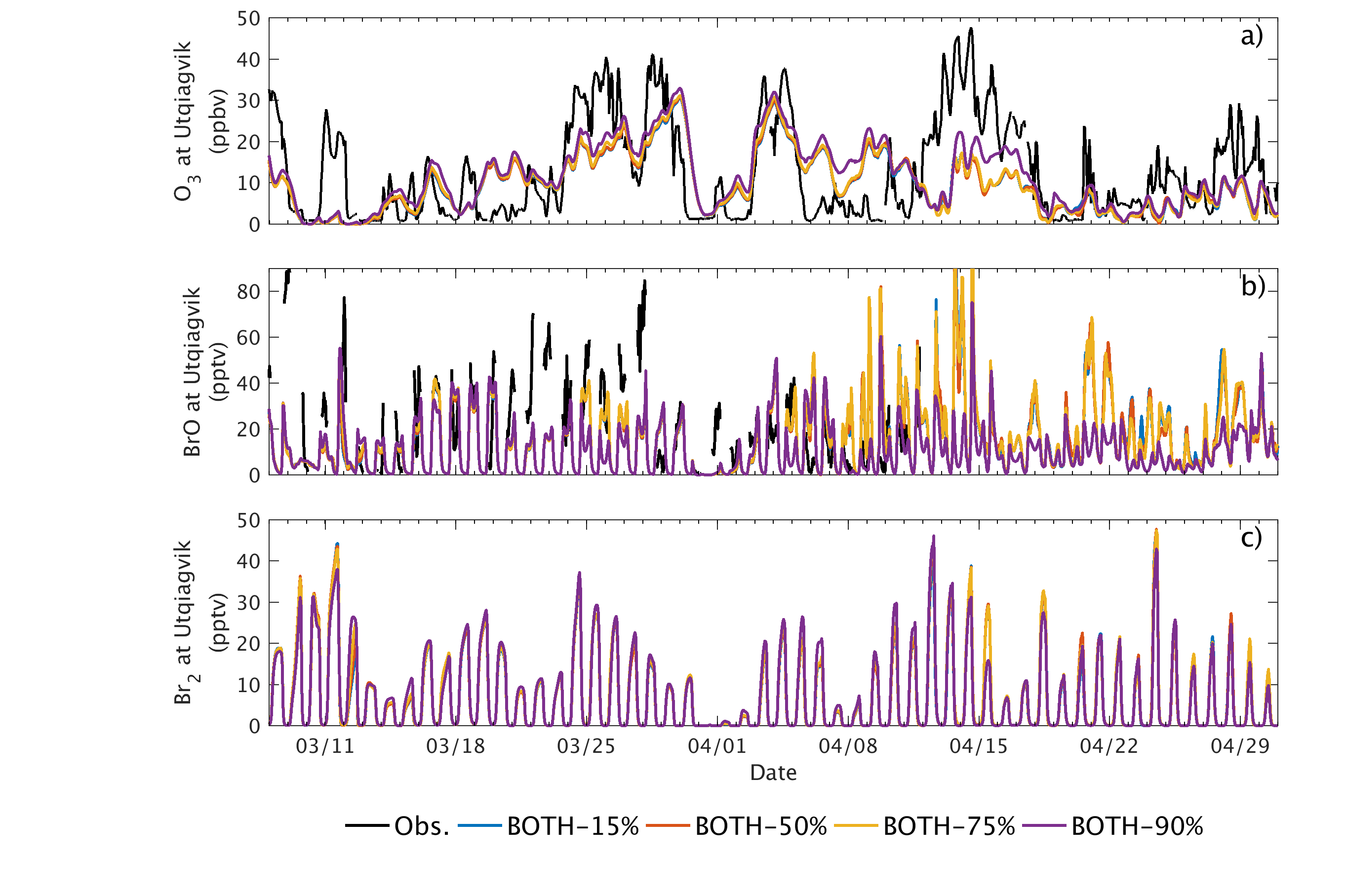
Figure S2. Top panel: O_3_ observed (black) at Utqiagvik, Alaska and simulated by WRF-Chem in different BOTH simulations using 15% (blue), 50% (red), 75% (yellow), and 90% (purple) fractional sea ice thresholds (described above). Middle panel: BrO observed by MAX-DOAS during the BROMEX campaign at Utqiagvik, and simulated by WRF-Chem. Bottom panel: Br_2_ simulated by WRF-Chem at Utqiagvik.


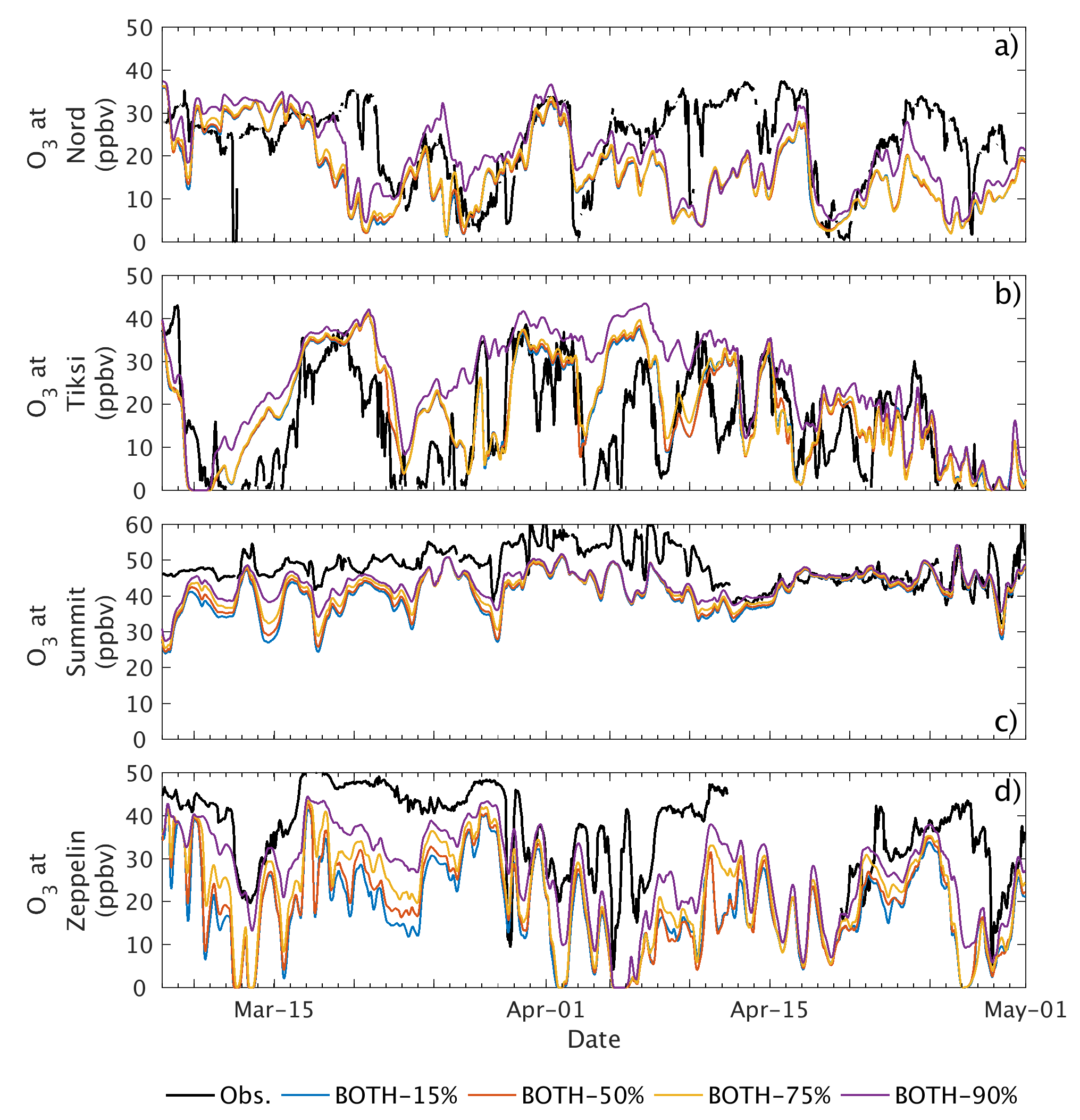


Figure S3. Surface ozone observed (black) and simulated by WRF-Chem for BOTH (color) simulations using 15% (blue), 50% (red), 75% (yellow), and 90% (purple) fractional sea ice thresholds at (a) Station Nord, Greenland; (b) Tiksi, Russia; (c) Summit, Greenland; and (d) Zeppelin Station, Svalbard. Compared to the main implementation presented in the manuscript, these simulations include blowing snow emissions with the original parameters from Yang et al. (2008), including a higher salinity (snow salinity distribution of mean 8.3 psu) and N=1 aerosol fractioning.


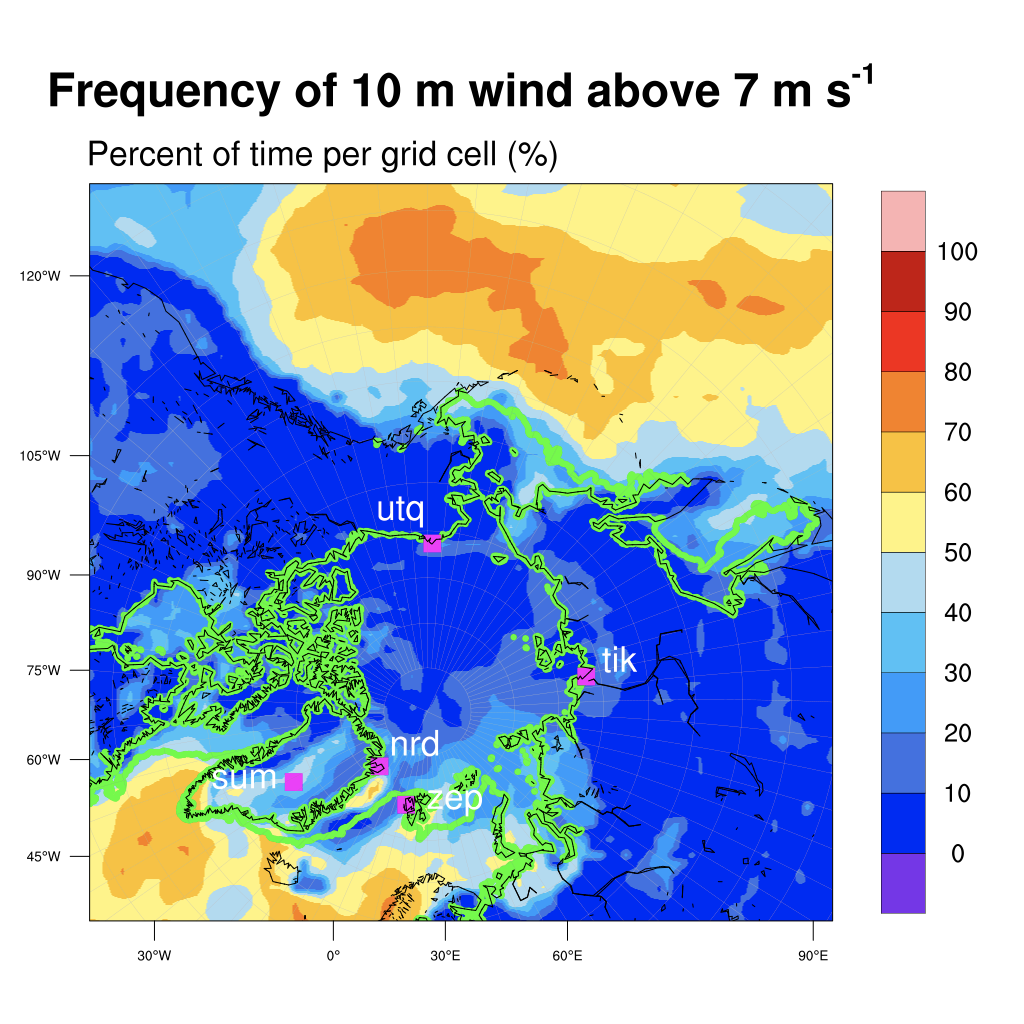


**Figure S4**. Percent (frequency of occurrence) of surface (10 m) wind speeds above 7 m/s predicted by WRF-Chem within each model grid cell (08/03/2012 - 01/05/2012). The sea ice extent from the Multisensor Analyzed Sea Ice Extent - Northern Hemisphere (MASIE-NH) is shown as the green line. The frequency of winds above 7 m/s (needed to loft blowing snow into the atmosphere) is below 10% over the sea ice found in the central Arctic Ocean. Surface stations used in the study are shown in magenta (utq = Utqiaġvik, nrd = Station Nord, sum = Summit, zep = Zeppelin, tik = Tiksi).


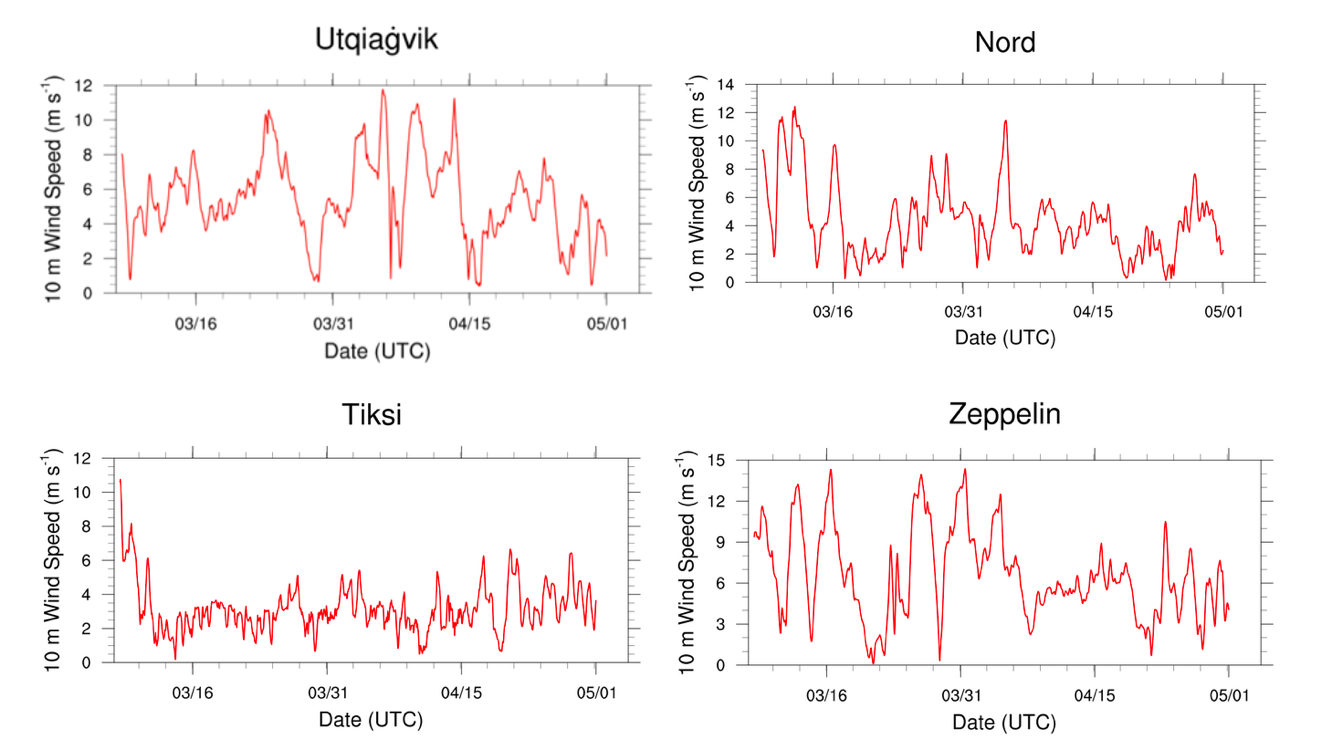


**Figure S5**. Surface wind speeds at the Arctic measurement sites used in this study. The threshold for lofting blowing snow is ~7 m/s.


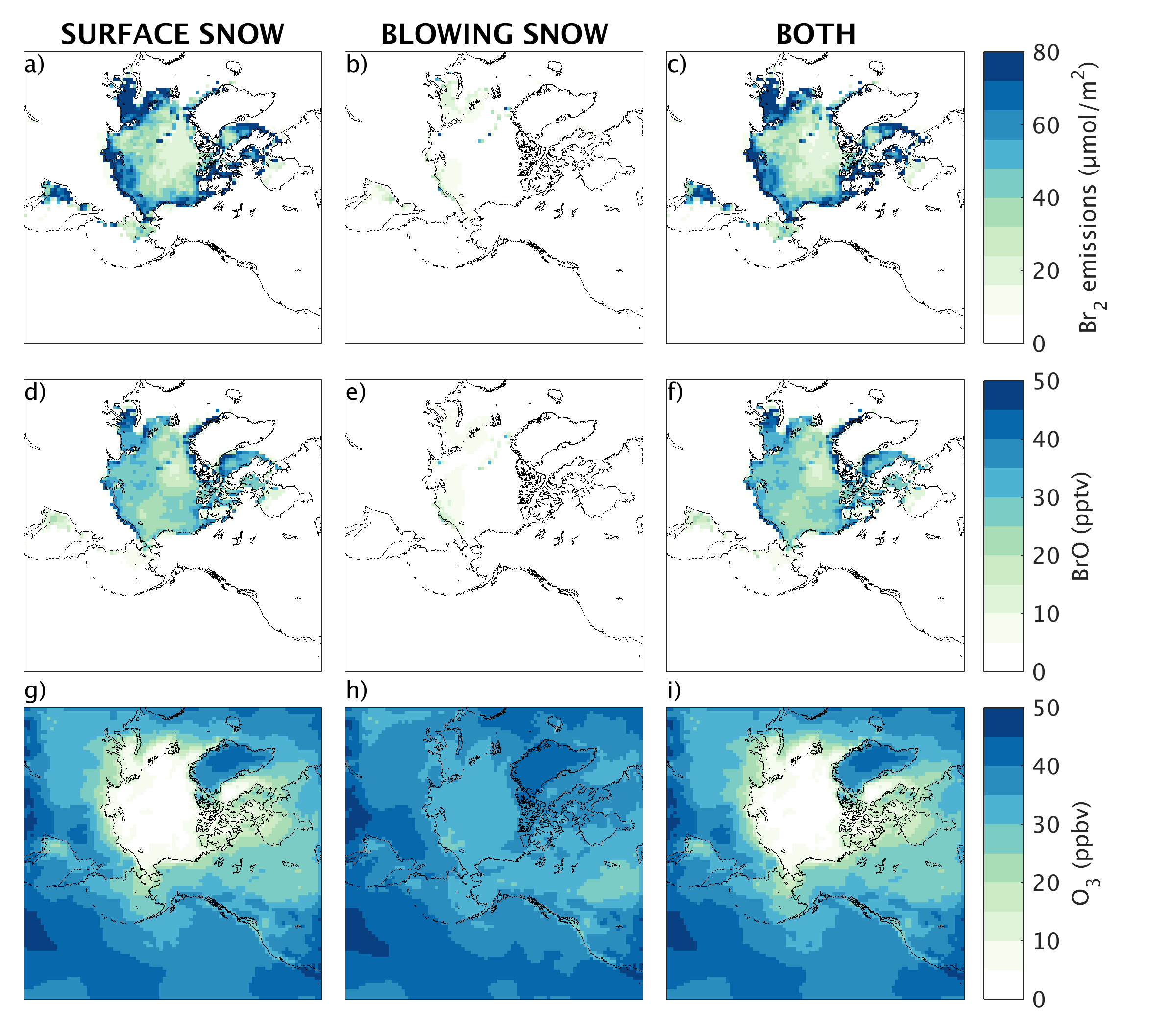


**Figure S6**. Monthly averaged (1 to 30 April 2012) modeled Br_2_ emissions (top), BrO concentrations (middle) and surface ozone concentrations (bottom), predicted across the Arctic for the SURFACE simulation (left panels), the BLOWING simulation (center panels), and the BOTH simulation (right panels).


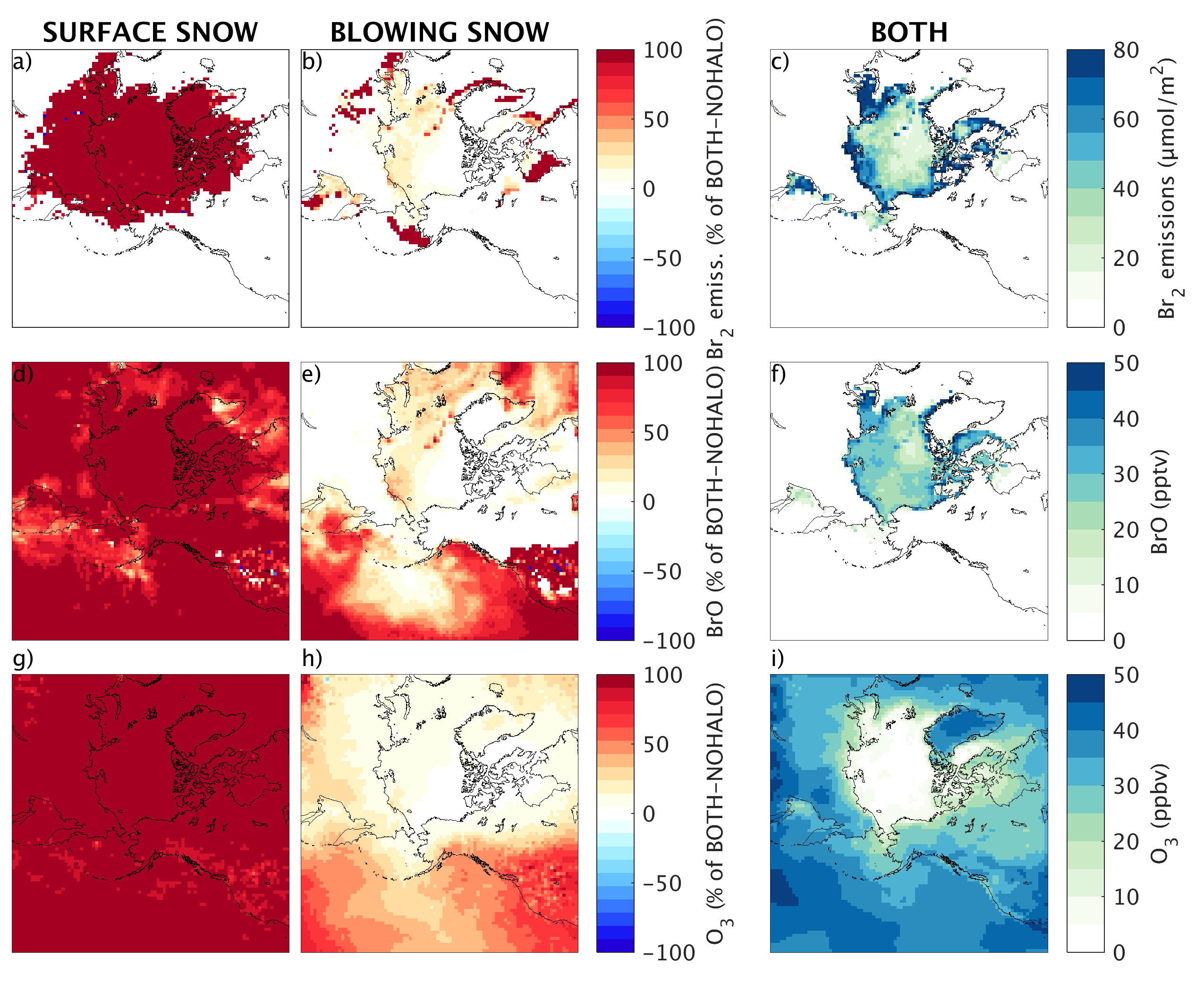


**Figure S7**. Monthly averaged (1 to 30 April 2012) relative increase in Br_2_ emissions and BrO and O_3_ concentrations in each simulation, compared to BOTH-NOHALO. Modeled Br_2_ emissions (top), BrO concentrations (middle) and surface ozone concentrations (bottom), predicted across the Arctic for (SURFACE-NOHALO)/(BOTH-NOHALO) (left panels), (BLOWING-NOHALO) )/(BOTH-NOHALO) (center panels), and absolute values in BOTH (right panels).


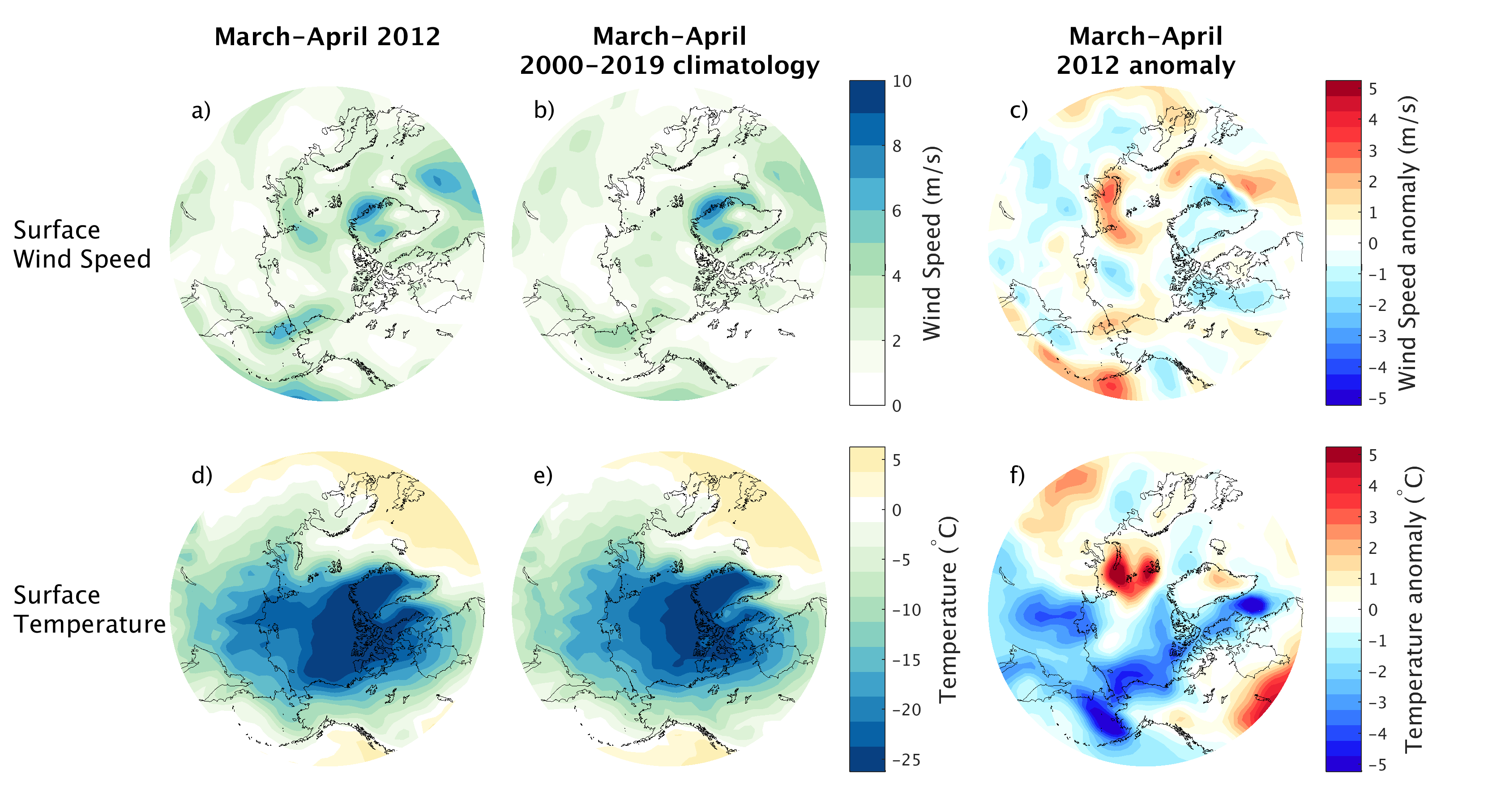


**Figure S8**. Surface wind speed (top) and temperature (bottom) climatologies and anomalies for March and April 2012. March and April 2012 average (left) compared to the March and April 2000-2019 average (middle) average from the NCEP reanalysis. The surface anomaly ([March_April 2012] - [March_April 2000-2019]) is also shown (right).


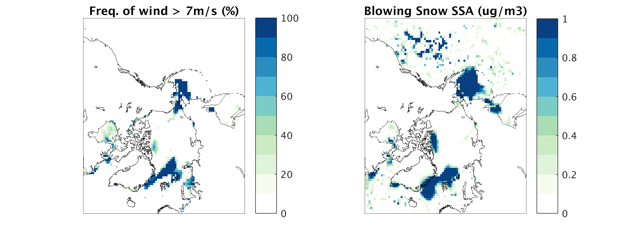


**Figure S9**. (Left) frequency of 10-m wind over 7 m/s (the approximate threshold for lofting snow) over sea ice during 1 March 2012. (Right) Daily averaged surface sea salt aerosol (SSA) concentration (0-10µm diameters) for the same day, showing the component from blowing snow only (BLOWING-NOHALO). Noise outside of the Arctic is due to random variability of rain and wet removal between BLOWING and NOHALO, which is more apparent for short time averages.


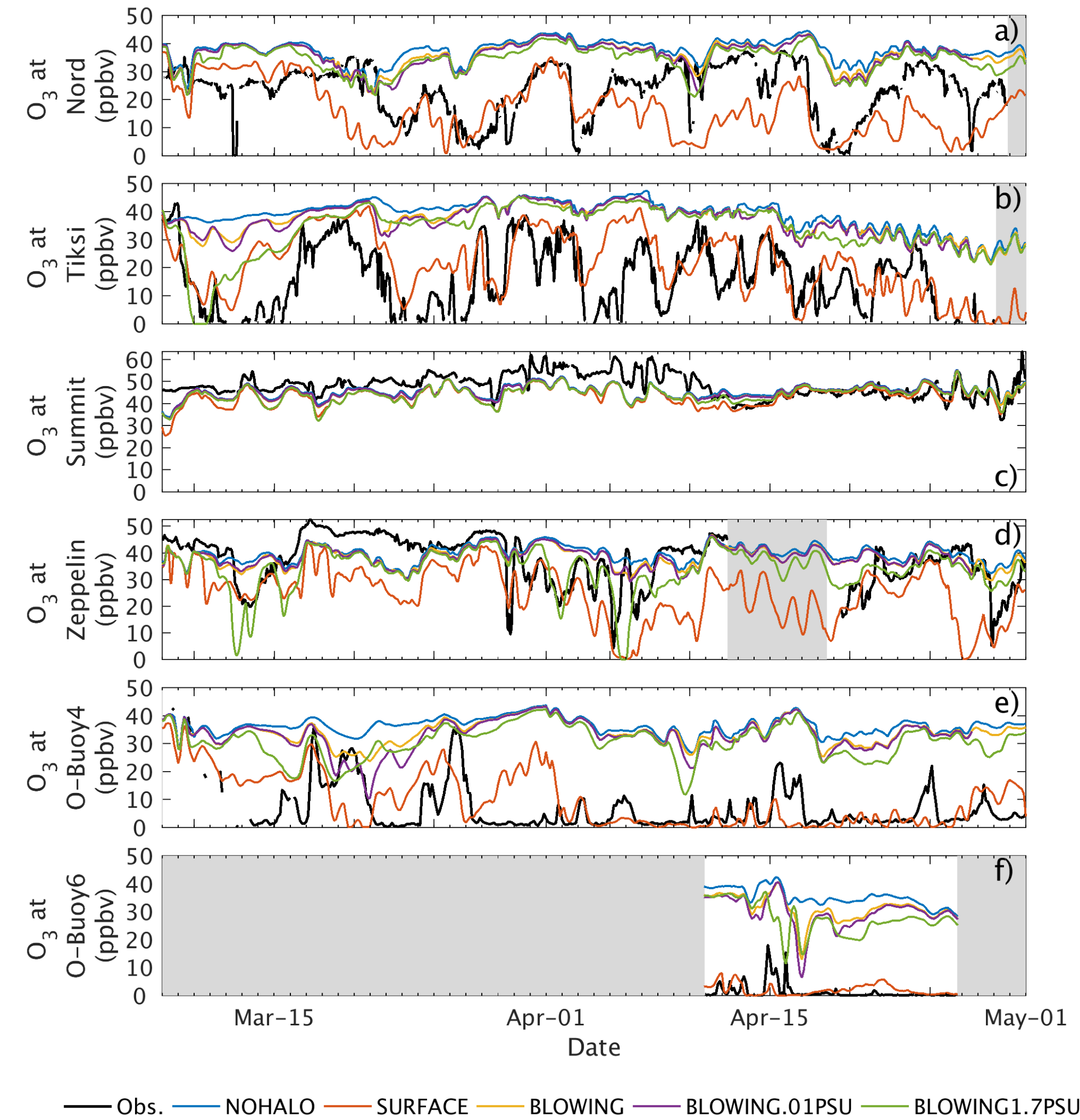


**Figure S10**. Surface ozone observed (black) and simulated by WRF-Chem (color) at (a) Station Nord, Greenland; (b) Tiksi, Russia; (c) Summit, Greenland; (d) Zeppelin Station, Svalbard; (e) O-buoy4, central Arctic; (f) O-buoy6, central Arctic. WRF-Chem simulations include the 3 main simulations from the manuscript (NOHALO, SURFACE, BLOWING), and 2 additional sensitivity blowing snow simulations using uniform snow salinities of 0.01 psu (BLOWING.01PSU) and 1.7 psu (BLOWING1.7 PSU).


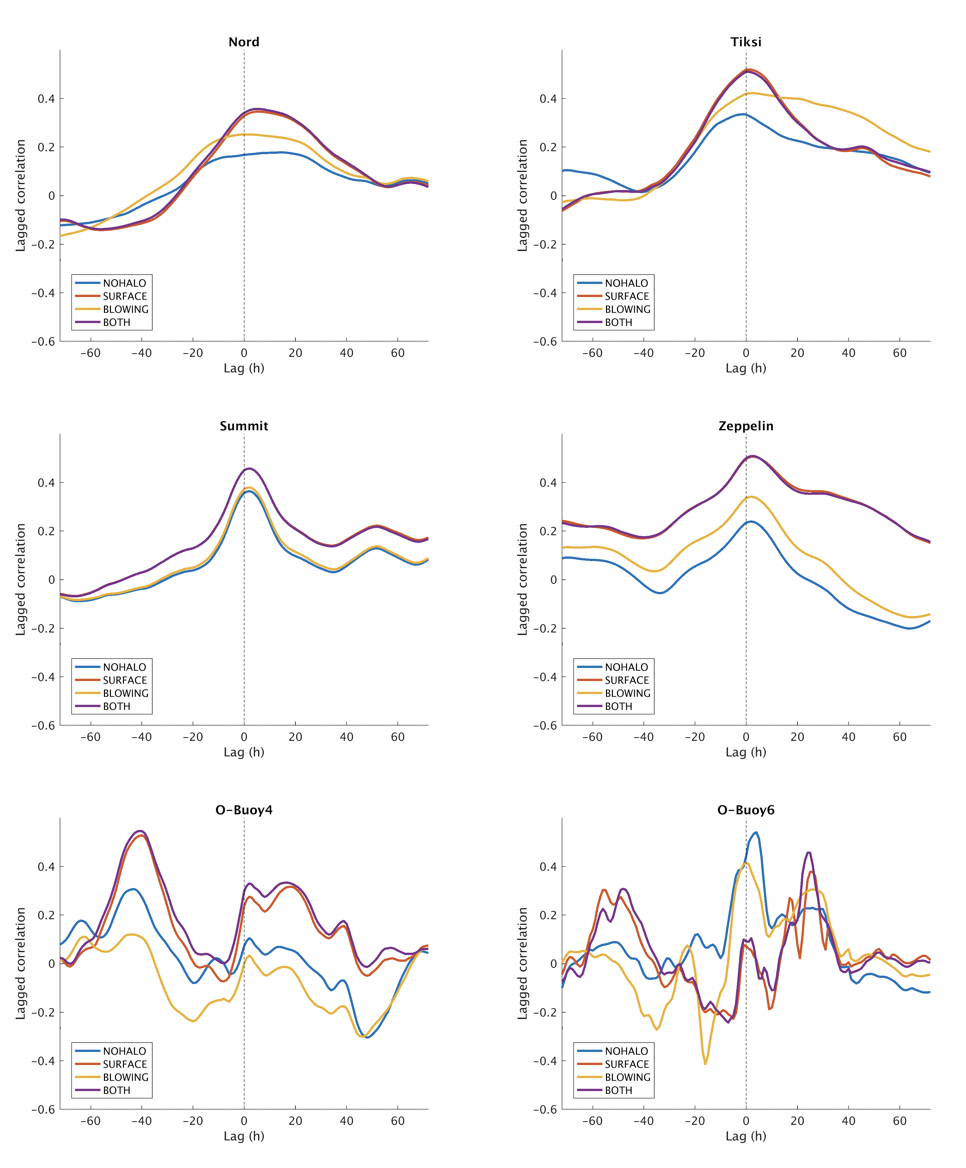


**Figure S11**. Time-lagged correlations (-72 h to 72 h) between observed and modeled ozone (NOHALO, SURFACE, BLOWING, BOTH) at the 6 Arctic sites.

Table S1. RMSE and correlation coefficient for each WRF-Chem simulation (8 March to 31 April 2012) compared to ground-based ozone measurements at the 5 surface sites and ozone measurements on 2 buoys in the Central Arctic.

| **Station name** |  | **NOHALO** | **SURFACE** | **BLOWING** | **BOTH** |
| --- | --- | --- | --- | --- | --- |
| Utqiagvik | *RMSE (ppbv)* | 25.4 | 10.3 | 23.4 | 10.3 |
|  | *R* | 0.22 | 0.50 | 0.20 | 0.50 |
| Nord | *RMSE (ppbv)* | 17.2 | 12.9 | 15.9 | 12.4 |
|  | *R* | 0.17 | 0.33 | 0.25 | 0.34 |
| Tiksi | *RMSE (ppbv)* | 24.6 | 12.1 | 22.7 | 12.3 |
|  | *R* | 0.33 | 0.52 | 0.42 | 0.51 |
| Summit | *RMSE (ppbv)* | 5.7 | 7.0 | 5.8 | 7.1 |
|  | *R* | 0.36 | 0.45 | 0.37 | 0.45 |
| Zeppelin | *RMSE (ppbv)* | 9.6 | 15.2 | 9.1 | 15.5 |
|  | *R* | 0.23 | 0.50 | 0.34 | 0.50 |
| O-buoy4 | *RMSE (ppbv)* | 30.9 | 10.5 | 28.8 | 10.4 |
|  | *R* | 0.07 | 0.24 | 0.0 | 0.30 |
| O-buoy6 | *RMSE (ppbv)* | 34.0 | 3.5 | 30.0 | 3.7 |
|  | *R* | 0.44 | 0.07 | 0.41 | 0.09 |
| 7 station mean | *RMSE (ppbv)* | 21.1 | 10.2 | 19.4 | 10.2 |
|  | *R* | 0.26 | 0.37 | 0.28 | 0.38 |
